# ADAM8 expression in invasive breast cancer promotes tumor dissemination and metastasis

**DOI:** 10.1002/emmm.201303373

**Published:** 2013-12-27

**Authors:** Mathilde Romagnoli, Nora D Mineva, Michael Polmear, Catharina Conrad, Srimathi Srinivasan, Delphine Loussouarn, Sophie Barillé-Nion, Irene Georgakoudi, Áine Dagg, Enda W McDermott, Michael J Duffy, Patricia M McGowan, Uwe Schlomann, Maddy Parsons, Jörg W Bartsch, Gail E Sonenshein

**Affiliations:** 1 Department of Developmental, Molecular and Chemical Biology, Tufts University School of MedicineBoston, MA, USA; 2 Department of Biomedical Engineering, Tufts UniversityMedford, MA, USA; 3 Department of Neurosurgery, Philipps UniversityMarburg, Germany; 4 UMR 892 INSERMNantes, France; 5 Education and Research Centre, St. Vincent's University HospitalDublin, Ireland; 6 Conway Institute of Biomolecular and Biomedical Research, UCD School of Medicine and Medical Science, University College DublinDublin, Ireland; 7 King's College LondonLondon, UK

**Keywords:** ADAM8, cancer progression, metastasis, therapeutic target, triple-negative, breast cancer

## Abstract

The transmembrane metalloprotease-disintegrin ADAM8 mediates cell adhesion and shedding of ligands, receptors and extracellular matrix components. Here, we report that ADAM8 is abundantly expressed in breast tumors and derived metastases compared to normal tissue, especially in triple-negative breast cancers (TNBCs). Furthermore, high *ADAM8* levels predicted poor patient outcome. Consistently, ADAM8 promoted an aggressive phenotype of TNBC cells in culture. In a mouse orthotopic model, tumors derived from TNBC cells with ADAM8 knockdown failed to grow beyond a palpable size and displayed poor vascularization. Circulating tumor cells and brain metastases were also significantly reduced. Mechanistically, ADAM8 stimulated both angiogenesis through release of VEGF-A and transendothelial cell migration via β1-integrin activation. *In vivo*, treatment with an anti-ADAM8 antibody from the time of cell inoculation reduced primary tumor burden and metastases. Furthermore, antibody treatment of established tumors profoundly decreased metastases in a resection model. As a non-essential protein under physiological conditions, ADAM8 represents a promising novel target for treatment of TNBCs, which currently lack targeted therapies and frequently progress with fatal dissemination.

**Subject Category** Cancer

## Introduction

Cancer metastasis results from a multistep process that selects for invasive tumor cells capable of escaping from the primary site and colonizing distant organs. Early in the tumorigenesis process, the hypoxic stress that characterizes the low tissue oxygen microenvironment of a solid tumor can stimulate tumor-induced angiogenesis, which in turn supplies nutrients and oxygen necessary to overcome tumor dormancy by supporting tumor growth, as well as providing routes for tumor cell dissemination (Harris, 2002; Majmundar *et al*, 2010). Secretion of pro-angiogenic mediators by tumor cells, most notably vascular endothelial growth factor A (VEGF-A), promotes endothelial cell migration and proliferation (Adams & Alitalo, 2007). The resulting neovascularization of the surrounding stroma facilitates the spreading of tumor cells into the blood stream, a process that requires adherence to and transmigration through the endothelium, in particular via processes involving integrins (Avraamides *et al*, 2008; Mierke, 2008). Circulating tumor cells (CTCs) can be measured in the peripheral blood even before the tumor is clinically detectable (Eyles *et al*, 2010). The CTC concentration constitutes a reliable parameter to identify breast cancer patients at high risk for disease recurrence and poor outcome (Cristofanilli *et al*, 2004). Despite recent advances in the treatment of cancer, the World Health Organization reports that over 500 000 women worldwide die yearly of metastatic breast disease. Identifying new therapeutic targets to reduce breast cancer dissemination is crucial to improve the overall survival of patients at high risk. In particular, women with triple-negative breast cancers (TNBCs), which are negative for estrogen receptor, progesterone receptor and HER2 overexpression, lack targeted therapy (Boyle, 2012).

ADAM8 is a transmembrane protein that belongs to the a disintegrin and metalloprotease (ADAM) family and mediates cell adhesion, cell migration, and proteolysis of various substrates, including cytokine receptors or their ligands, cell adhesion molecules and extracellular matrix components (for review Koller *et al*, 2009). Synthesized as a proform, ADAM8 can dimerize or multimerize and autocatalytically clip off its prodomain, leaving an active membrane-anchored metalloprotease. Active ADAM8 can be further processed by the release of the metalloprotease domain into the extracellular matrix, which leaves a remnant form within the membrane. Both active and remnant forms mediate cell adhesion through their disintegrin/cysteine-rich/EGF-like domains (Schlomann *et al*, 2002), notably by direct binding to integrins (Rao *et al*, 2006). ADAM8 was found to be non-essential under physiological conditions, as evidenced by normal development, lack of pathological defects, and a normal life span of ADAM8 deficient mice (Kelly *et al*, 2005; Bartsch *et al*, 2010), despite its expression in a variety of immune cells (Yoshiyama *et al*, 1997; Richens *et al*, 2007). Interestingly, ADAM8 expression was detected under several pathological conditions characterized by inflammation and extracellular matrix remodeling, including cancer (Koller *et al*, 2009). Recently, using microarray analysis, we identified ADAM8 as a target of a multistep pathway downstream of NF-κB RelB, which promotes an aggressive phenotype in breast cancer (Wang *et al*, 2007). Here, our analysis of publicly available microarray databases indicated that *ADAM8* is overexpressed in aggressive breast cancers, including TNBCs, and correlated with a poor patient outcome. As ADAM8 was required to maintain the aggressive phenotype of TNBC cells in 3D-culture, we further investigated its role in breast cancer pathology. Using both knockdown and antibody targeting strategies, ADAM8 was shown to promote TNBC tumor growth, angiogenesis, spread of CTCs and metastatic dissemination in orthotopic mouse models. Our findings validate the transmembrane ADAM8 protein as a promising novel target for the treatment of these aggressive breast tumors.

## Results

### High ADAM8 expression in human breast tumors correlates with poor prognosis

**Figure 1:**
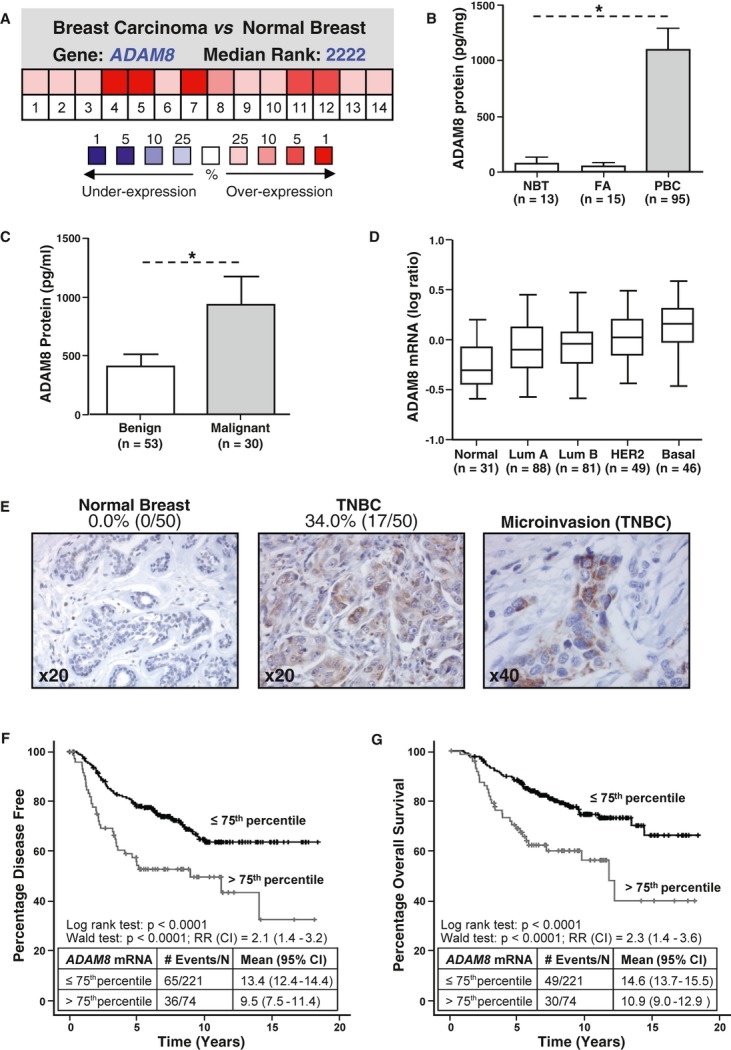
A *ADAM8* mRNA expression in samples from breast tumor and normal breast tissue was analyzed using the Oncomine microarray database. Pooling of 14 analyses from six different microarray studies shows *ADAM8* is one of the more highly expressed genes in breast cancer versus normal tissue. *P *= **0.025, Student's *t*-test. B, C ADAM8 protein levels were measured by ELISA in samples from adjacent normal breast tissue (NBT), fibroadenoma (FA) and primary breast carcinoma (PBC) (B), and in serum of patients with either benign or malignant breast disease (C). * *P *< **0.0001, Kruskal–Wallis test (B); * *P *= **0.034, Mann–Whitney *U*-test (C). D *ADAM8* mRNA expression was analyzed across the different molecular breast cancer subtypes in the van de Vijver microarray dataset, which includes 295 primary breast tumors from normal-like (Normal), luminal A (Lum A), luminal B (Lum B), HER2, and basal-like (Basal) subtypes (van de Vijver *et al*, 2002). * *P *< **0.0001 for Basal versus other groups, Kruskal–Wallis test. E Representative pictures of ADAM8 staining analyzed by immunohistochemistry in adjacent normal epithelial tissue and primary TNBC samples from 50 patients or areas of microinvasion. Percentages of ADAM8-positive samples are given. F, G Kaplan–Meier curves show the percentage of disease-free survival (F) and overall survival (G) for 295 patients with primary breast cancer stratified based on *ADAM8* mRNA levels using the 75th percentile. *P *< **0.0001, Log-rank test (RR, relative risk, CI, confidence interval).

Using the Oncomine microarray database to assess *ADAM8* mRNA levels in breast cancer, *ADAM8* was identified as one of the more highly expressed genes in human breast tumors in comparison to normal breast tissue (Fig 1A). Consistently, ADAM8 protein levels were strikingly higher in primary breast tumor tissue compared to either adjacent normal mammary tissue or fibroadenomas, which are the most common benign tumors of the breast (Fig 1B). Serum levels of ADAM8 protein were also significantly higher in patients with breast cancer compared to those with benign disease (Fig 1C). Of interest, basal-like breast carcinomas, which are typically highly aggressive and mostly TNBC (Bertucci *et al*, 2012), expressed the highest levels of *ADAM8* mRNA compared to normal-like, luminal A and B, or HER2-overexpressing breast cancers (Fig 1D). Immunohistochemical analysis of breast tumors demonstrated that ADAM8 was localized to the cytoplasm and plasma membrane of cancer cells, and was abundantly observed in 34.0% of TNBCs (Fig 1E). Interestingly, ADAM8 expression was detected at the leading front of microinvasive areas at primary tumor sites (Fig 1E, right panel). In contrast, ADAM8 was not detectable in adjacent normal mammary tissue of TNBCs (Fig 1E, left panel). In addition, only 13.5% (5/37) of ductal carcinoma *in situ* (DCIS) tumors, which are defined by the lack of local invasion out of the mammary ducts, were positive for ADAM8 staining.

Analysis of the van de Vijver *et al* (2002) microarray dataset revealed *ADAM8* mRNA levels were higher in tumors > 2 cm in diameter compared to those with a diameter of less than 2 cm, and in grade 3 tumors compared to those with lower grades (supplementary Fig S1A and B). In Kaplan–Meier curves, high *ADAM8* mRNA levels significantly correlated with poor disease-free and overall survival in the total patient population (Fig 1F and G) or when the 41 patients with basal tumors were removed (overall survival using 75th percentile cutoff in the dataset minus basal samples: *P *= **0.003, HR = 2.41, CI = 1.36–4.27). In a multivariate analysis that included tumor size, tumor grade, lymph node status and ER status, *ADAM8* mRNA level was found to be an independent predictor of poor disease-free ( *P *= **0.001) and overall survival ( *P *= **0.052) (supplementary Table S1). Together, these findings demonstrate that ADAM8 is aberrantly expressed in a large number of breast cancers, in particular in TNBCs, and its expression is associated with a worse prognosis. Thus, we examined the role of ADAM8 in TNBCs, which are characterized by high rates of recurrence and dissemination to distant organs (Boyle, 2012).

### ADAM8 expression in TNBC cells is increased in 3D suspension cultures

**Figure 2:**
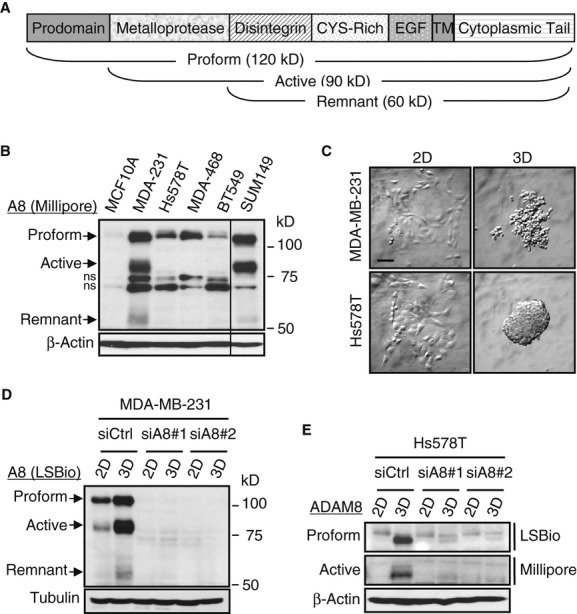
A Schematic representation of ADAM8 protein with its domains, processed forms and molecular weights indicated. CYS-Rich: cysteine-rich, EGF: EGF-like, TM: transmembrane domains. B Whole-cell extracts (WCEs) from human non-tumoral MCF-10A cells and the indicated TNBC cell lines were examined by WB for ADAM8 expression (Millipore antibody), and for β-Actin as a loading control. A representative blot is shown ( *n* = 3). All lanes were from the same gel, but cut to re-align as indicated by the vertical line. ADAM8 forms and MW markers are indicated. ns: non-specific band. MDA-231: MDA-MB-231. MDA-468: MDA-MB-468. C Cells were grown in adherent (2D) or suspension cultures on ultra low-attachment plates (3D) for 48 h. In suspension, MDA-MB-231 and Hs578T cells form spheres. Bar: 100 μm. D, E MDA-MB-231 (D) and Hs578T (E) cells were transfected with either *Control* siRNA (siCtrl) or two specific *ADAM8* siRNAs (siA8) for 24 h. Cells were then plated in 2D-or 3D-culture as described in (C). WCEs were subjected to WB for ADAM8 using only LSBio antibody (D), or using both LSBio and Millipore antibodies (E). As a loading control, Tubulin (D) or β-Actin (E) was used. Representative blots are shown ( *n* = 3).

ADAM8 is synthesized as a 120 kD proform, which multimerizes and autocatalytically clips off its prodomain, leaving a proteolytically active 90 kD membrane-anchored metalloprotease, which can be further processed to a 60 kD remnant form (Fig 2A) (Koller *et al*, 2009). Using western blotting (WB), high ADAM8 proform expression was found in all TNBC cell lines tested compared to the non-tumoral mammary epithelial cell line MCF-10A when grown in adherent conditions (Fig 2B). Processed active and remnant ADAM8 forms were prominent in MDA-MB-231 and SUM149 cells. Growth of MDA-MB-231 and Hs578T cells under low attachment conditions, which selects for a more aggressive phenotype, was used to further study cells with or without evident active processing in 2D-culture. Both cell lines formed spheres when grown in 3D suspension culture (Fig 2C). In MDA-MB-231 cells, robust increases in all ADAM8 forms were seen with 3D-culture (Fig 2D). Interestingly, when Hs578T cells were grown in 3D-culture conditions, substantial levels of active form were now observed, as well as higher levels of ADAM8 precursor proform (Fig 2E). We also noted that the ADAM8 proform in Hs578T spheres displayed faster migration, similar to the band observed in MDA-MB-231 cells (supplementary Fig S2), which may be due to altered posttranslational modifications. The precursor and active ADAM8 forms in Hs578T cells were better detected using LSBio and Millipore antibodies, respectively. Use of two different specific *ADAM8* siRNAs led to effective ADAM8 knockdown (KD) in the two lines under both growth conditions (Fig 2D and E). Thus, ADAM8 is expressed and processed to an active form in TNBC cells, and its levels increase when cells are grown in suspension as ‘tumorspheres’.

### TNBC cell migratory and invasive properties are maintained by ADAM8

**Figure 3:**
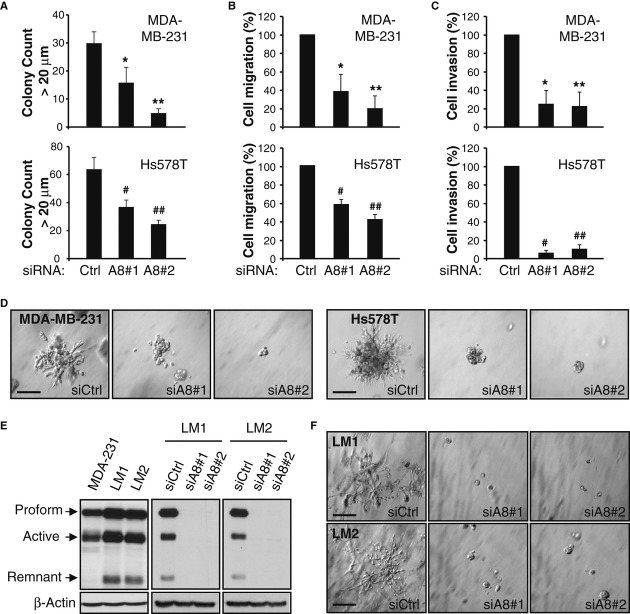
A–D Cells were transfected with siRNAs as in Fig 2D and E for 24 h, and tested for colony formation in soft agar (A), cell migration (B), invasion (C), and 3D-Matrigel outgrowth (D). For soft agar assays, cells were grown for 8-12 days and colonies >20 μm diameter in 3 wells/condition were counted with ImageJ software. A representative of two experiments with similar results is shown. * *P *= **0.002, ** *P *= **1.9E-6, ^#^ *P *= **1.3E-5, ^##^ *P *= **1.0E-7, Student's *t-*test (A). Migration (B) and invasion (C) assays were performed for 24 h using Transwells without or with precoating of Matrigel, respectively. Control condition (siCtrl) is set to 100% (mean ± s.d. from three independent experiments). * *P *= **0.004, ** *P *= **5.3E-4, ^#^ *P *= **1.8E-4, ^##^ *P *= **5.5E-5, Student's *t-*test (B). * *P *= **0.001, ** *P *= **9.3E-4, ^#^ *P *= **8.7E-6, ^##^ *P *= **4.6E-5, Student's *t-*test (C). For Matrigel outgrowth assays, colonies formed after 5-7 days were photographed at 20× magnification. Experiments were done twice with similar results. Bars: 100 μm (D). E WCEs from MDA-MB-231 cells and *in vivo* derived LM1 and LM2 lines, either untransfected (left panel) or transfected with siCtrl or siA8 for 48 h (right panels), were subjected to WB for ADAM8 (LSBio antibody). A representative blot is shown ( *n* = 3). F Twenty-four h after siRNA transfection, LM1 and LM2 cells were subjected to a 3D-Matrigel outgrowth assay, as in (D), except that colonies were photographed after 4 days. Experiments were done twice with similar results. Bars: 100 μm.

To test the role of ADAM8 in the aggressive phenotype of MDA-MB-231 and Hs578T cells *in vitro*, the effects of its KD were measured on growth in soft agar, migration and invasion through Matrigel, assays that are performed in 3D-culture conditions. Decreased ADAM8 expression in both lines led to reduced abilities to grow under anchorage-independent conditions (Fig 3A), to migrate (Fig 3B) or invade through Matrigel (Fig 3C). Consistently, the capacity of these aggressive cells to form characteristic branching colonies in a Matrigel 3D-outgrowth assay was robustly inhibited upon ADAM8 KD (Fig 3D). The MDA-MB-231-derived cells LM1 and LM2, which were selected *in vivo* for their ability to metastasize to the lung (Minn *et al*, 2005), expressed higher levels of ADAM8 than the parental line (Fig 3E), and formed large colonies more rapidly in Matrigel (3–4 versus 6–7 days for parental cells) (Fig 3F). The ability of LM1 and LM2 cells to form these highly invasive colonies in Matrigel was strikingly reduced by ADAM8 KD (Fig 3E and F). Thus, ADAM8 is required to maintain the migratory and invasive phenotype of TNBC cells in culture.

### ADAM8 promotes tumor growth, spreading of CTCs and metastasis

**Figure 4:**
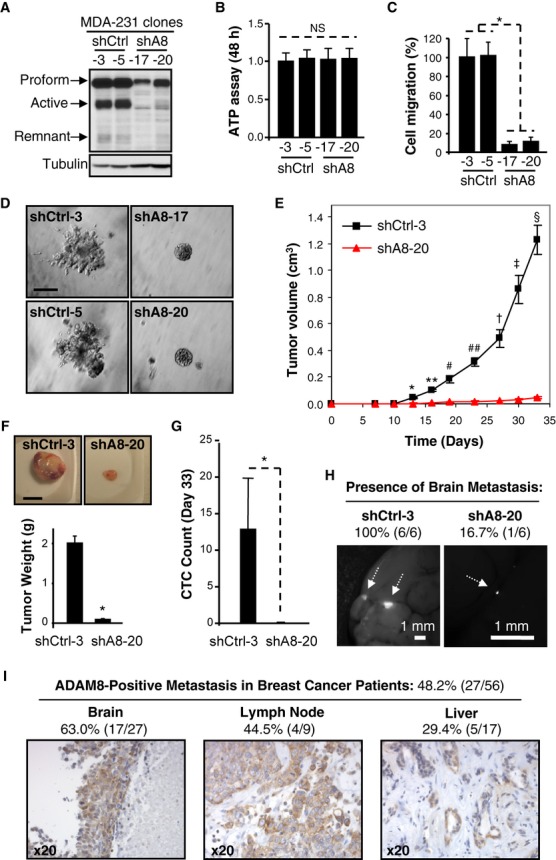
A–D Stable ADAM8 KD (shA8) clones were characterized *in vitro*. ADAM8 expression in WCEs from two shA8 clones (shA8-17 and shA8-20) were compared with two shCtrl clones (shCtrl-3 and shCtrl-5) using WB (LSBio antibody) (A). Clones were grown in 2D-cultures for 48 h and subjected to an ATP assay. NS: non significant, Student's *t*-test, *n* = 3 (B). Migration assays were performed as described in Fig 3B. * *P *= **6.8E-14, Student's *t-*test, *n* = 3 (C). Matrigel outgrowth assays were performed as in Fig 3D. Experiments were done twice with similar results. Bar: 100 μm (D). E–H MDA-MB-231 derived shCtrl-3 and shA8-20 cells were injected into the MFP of female mice ( *n* = 7/group). Tumor volume was measured twice a week (mean ± s.e.m.). * *P *= **1.4E-6, ** *P *= **9.8E-8, ^#^ *P *= **3.0E-5, ^##^ *P *= **9.7E-7, ^†^ *P *= **1.7E-5, ^‡^ *P *= **5.1E-6, ^§^ *P *= **2.0E-7, Student's *t*-test (E). At the end of the experiment, tumors were photographed and weighed (mean ± s.e.m.). * *P *= **5.6E-8, Student's *t*-test. Bar: 1 cm (F). Blood was collected by cardiac puncture and GFP-positive CTCs were detected by flow cytometry. Average CTC count ± s.d. is given per μl of blood. * *P *= **0.006, Student's *t*-test (G). Presence of brain metastases was examined by fluorescent microscopy ( *n* = 6/group). Representative photographs are shown. Bars: 1 mm (H). I ADAM8 expression in distant metastases of breast cancer patients ( *n* = 56) was analyzed by immunohistochemistry. Representative pictures and percentages of ADAM8-positive samples are presented.

Next, we tested the effects of stable ADAM8 KD *in vivo* using a mouse mammary fat pad (MFP) xenograft model. Clones of MDA-MB-231 cells expressing a specific *ADAM8* shRNA (shA8-17 and shA8-20) or *Control* shRNA (shCtrl-3 and shCtrl-5) were isolated. Effective KD of ADAM8 was confirmed by WB (Fig 4A). Reduction of ADAM8 had no detectable effect on 2D-growth as assessed by an ATP assay (Fig 4B). The two shA8 MDA-MB-231 clones showed significantly reduced ability both to migrate and to invade through Matrigel compared to the shCtrl clones (Fig 4C and D). Rescue of these phenotypes by ectopic ADAM8 expression in the shA8-20 clone confirmed the role of ADAM8 KD in these cells (supplementary Fig S3).

To test the effects of ADAM8 KD on tumor growth and metastasis, MDA-MB-231 shCtrl-3 and shA8-20 cells were injected into the MFP of female NOD/SCID mice ( *n* = 7/group). Tumor growth at the orthotopic site was recorded twice weekly. About 2 weeks after cell implantation, mice from the shCtrl group started to develop mammary tumors that progressed rapidly (Fig 4E). In contrast, tumors derived from the shA8-20 clone failed to grow beyond 0.05 cm^3^ even after more than 4 weeks (day 33). The experiment was terminated when tumors in the shCtrl group reached a size of approximately 1 cm^3^ as per the approved mouse protocol. The tumors were harvested, photographed and weighed (Fig 4F). A dramatic decrease in average tumor weight was measured (2.00 ± 0.08 versus 0.16 ± 0.01 g). Visually, the tumors derived from the shA8-20 clone appeared to be substantially less vascularized (supplementary Fig S4).

CTCs were detected based on stable GFP expression in the MDA-MB-231 clones, using flow cytometry on freshly collected blood at the end of the experiment. A profound decrease in the numbers of CTCs was observed with ADAM8 KD (12.8 ± 7.0 versus 0.0 ± 2.0/μl of blood) (Fig 4G), suggesting that ADAM8 might be involved in dissemination of CTCs; although the possibility that this relates to the large difference in tumor burden could not be excluded. This question is further addressed below. To test whether KD of ADAM8 affects metastasis, we examined isolated brains ( *n* = 6/group) under a fluorescent dissecting microscope for the presence of GFP-positive metastases. All of the animals injected with shCtrl cells were positive for brain metastases, while only one mouse was detected with a small metastasis in the shA8 group (Fig 4H). This led us to investigate ADAM8 expression levels in metastases from breast cancer patients. Immunohistochemical analysis of 56 human breast cancer-derived metastases revealed that 48.2% (27/56) were positive for ADAM8 expression (Fig 4I), including the majority of brain metastases (63%), and significant percentages of lymph nodes (44.5%), liver (29.4%) and other organ metastases (33.3%, 1/3). In summary, ADAM8 is essential for the growth and spread of MDA-MB-231 cell-derived mammary tumors in an orthotopic xenograft model and consistently, ADAM8 expression is detected in almost half of distant metastases in patients with breast cancer.

### Knockdown of ADAM8 induces angiogenic tumor dormancy

**Figure 5:**
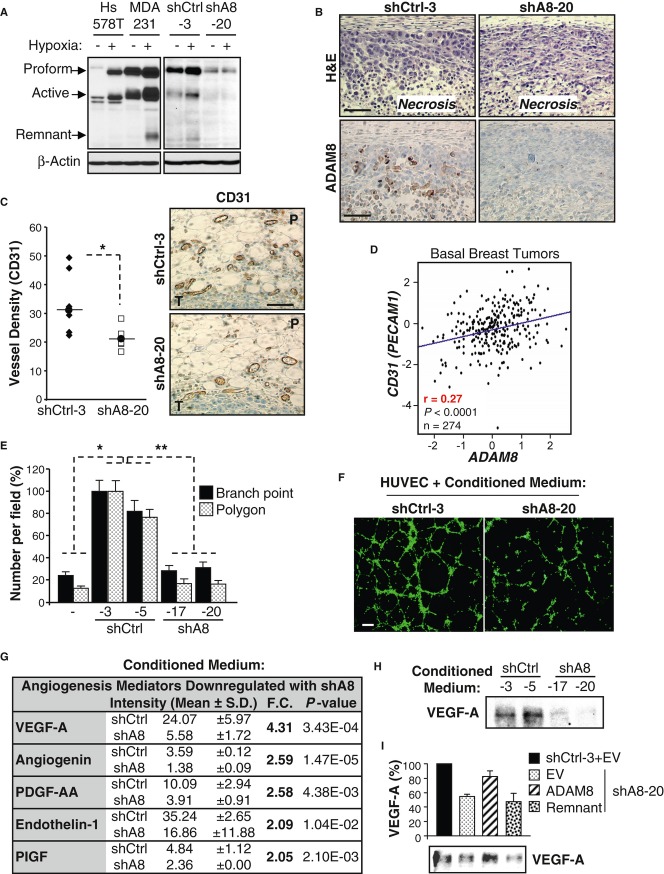
A Sixteen h after plating, cells were cultured under normoxic (−) or hypoxic (+, 1% O2) conditions for 24 h (MDA-MB-231 and Hs578T) or 6 h (shCtrl-3 and shA8-20 clones). WCEs were subjected to WB for ADAM8 (LSBio antibody). Representative blots are shown ( *n* = 3). B ADAM8 expression in mouse mammary tumors derived from shCtrl-3 or shA8-20 cells was analyzed by immunohistochemistry. H&E staining was performed in parallel. Representative panels are shown ( *n* = 7/group). Bar: 100 μm. C Angiogenesis was evaluated by CD31 immunohistochemical staining of tumor sections from shCtrl-3 and shA8-20 groups ( *n* = 7/group). Vessel density for each mouse is given as the average number of vessels in 2 slides/tumor (3 peritumoral hot spots/slide). P: Peritumoral area, T: Tumor. Bar: 100 μm. * *P *= **0.01, Student's *t*-test. D Pearson's pairwise correlation plot shows a significant positive correlation between *ADAM8* and *CD31 (PECAM1)* mRNA expression in tumors from patients with basal-like breast cancer (GenExMiner microarray database). r: correlation ratio. *P *< **0.0001, Student's *t*-test. E, F HUVECs were subjected to tube formation assays in the presence of conditioned medium from the indicated shA8 and shCtrl clones, or obtained in absence of tumor cells (−). Values for branch points and closed networks (polygons) are given as averages of nine fields ± s.d. Branch point: * *P *= **1.3E-6 versus shCtrl-3, * *P *= **3.2E-9 versus shCtrl-5, ** *P *= **6.9E-29; Polygons: * *P *= **5.2E-6 versus shCtrl-3, * *P *= **1.3E-6 versus shCtrl-5, ** *P *= **6.2E-24; Student's *t*-test; *n* = 4 (E). Representative images from 4 independent experiments are shown. Bar: 30 μm (F). G Conditioned media from two shCtrl and two shA8 clones were subjected to a Human Angiogenesis antibody array. Expression levels of the detected proteins were quantified using ImageJ and the angiogenesis mediators significantly downregulated by more than 2-fold in shA8 clones are presented as mean of the two clones ± s.d. Fold change (F.C.) and *P*-values are given, Student's *t*-test. H Conditioned serum-free medium from shCtrl and shA8 clones was analyzed by WB for VEGF-A. A representative blot is shown ( *n* = 3). I VEGF-A in the conditioned serum-free medium from shCtrl-3 or shA8-20 clones transfected with the indicated ADAM8 forms or empty vector (EV) DNA was assessed by WB (lower panel). The quantification of average levels from 3 experiments is presented as percent relative to the shCtrl set to 100%.

When solid tumors reach a few millimeters in diameter, nutrients and oxygen become insufficient and hypoxic stress is induced (Majmundar *et al*, 2010). This leads to cell death by necrosis as well as to the release of factors that promote endothelial cell recruitment and angiogenesis that are needed to support continued tumor growth (Bergers & Benjamin, 2003; Naumov *et al*, 2006). ADAM8 levels were induced under hypoxia in pancreatic cancer cells (Valkovskaya *et al*, 2007). Thus, the effects of growth under reduced oxygen on ADAM8 expression in breast cancer cells were studied. Incubation of MDA-MB-231 and Hs578T cells in 1% oxygen led to substantial increases in ADAM8 protein levels (Fig 5A, left panels). As seen with 3D-culture of Hs578T cells, levels of the ADAM8 proform and active form increased, and the proform now co-migrated with the band observed in MDA-MB-231 cells. Similar to the MDA-MB-231 parental cell line, ADAM8 expression was increased in shCtrl-3 cells under hypoxic conditions; whereas no induction was seen in the shA8-20 clone (Fig 5A, right panels). The typical hypoxia-inducible factor 1-alpha (HIF-1α) induction was essentially comparable in these cells (supplementary Fig S5), suggesting that the hypoxic pathway is not impaired in the clones. Consistently, strong ADAM8 staining was detected around the necrotic areas in shCtrl-3 tumors, but absent in tumors derived from shA8-20 cells (Fig 5B), even though the extent of necrosis was similar in both tumor populations.

To assess angiogenesis, immunohistochemical staining of tumors for the endothelial cell marker CD31 was performed. A significant decrease in the number of peritumoral vessels was measured in shA8-20 versus shCtrl-3 tumors (Fig 5C). Consistent with the notion that tumors with higher ADAM8 levels are characterized by increased angiogenesis, a positive correlation was found between *ADAM8* and *CD31 (PECAM1)* mRNA expression in tumor samples from patients with basal-like breast cancer (Fig 5D). Together, these findings indicate that in the absence of ADAM8, MDA-MB-231-derived tumors underwent angiogenic dormancy and were thus unable to grow past a palpable size.

### ADAM8 is necessary for release of pro-angiogenic factors

To determine the mechanism whereby ADAM8 induces angiogenesis, we first assessed the ability of conditioned media from MDA-MB-231 clones to stimulate the formation of vessel-like structures (tube formation) by human umbilical vein endothelial cells (HUVECs) on Matrigel (Fig 5E and F). The shCtrl cells released factors that substantially increased the number of branching points and polygons formed by HUVECs compared to control media. In contrast, tube formation was severely reduced in the presence of conditioned medium from shA8 clones (Fig 5E), indicating that ADAM8 promotes angiogenesis in part via a soluble factor.

To identify pro-angiogenic factors released by ADAM8, conditioned media from the two shCtrl and two shA8 MDA-MB-231 cell clones were analyzed using a Human Angiogenesis antibody array, which can detect the expression of 55 angiogenesis related proteins. Levels of the detected proteins were quantified and those that were significantly downregulated by more than an average of 2-fold in the shA8 clones are presented (Fig 5G). ADAM8 KD led to substantial decreases in five important angiogenesis mediators (VEGF-A, angiogenin, platelet-derived growth factor PDGF-AA, endothelin-1 and platelet growth factor PlGF), with the largest change seen in VEGF-A. To validate these results, we examined the expression of VEGF-A, a major inducer of angiogenesis, using a WB approach. A substantially decreased level of VEGF-A was seen in media of shA8 versus shCtrl cells (Fig 5H). Quantification from this and two additional independent experiments indicated that MDA-MB-231 cells released between 60 and 70% less VEGF-A ( *P *= **0.002) into the media when ADAM8 is knocked down. Notably, release of VEGF-A from the shA8 cells could be restored by ectopic expression of full-length ADAM8 but not by the remnant form (Fig 5I and supplementary Fig S6), indicating that the metalloproteinase domain was required. Thus, in the absence of ADAM8, less VEGF-A and other pro-angiogenic factors are released, which can account for the reduction of angiogenesis observed *in vivo*.

### ADAM8 promotes endothelial cell adhesion via activation of β1-integrin

**Figure 6:**
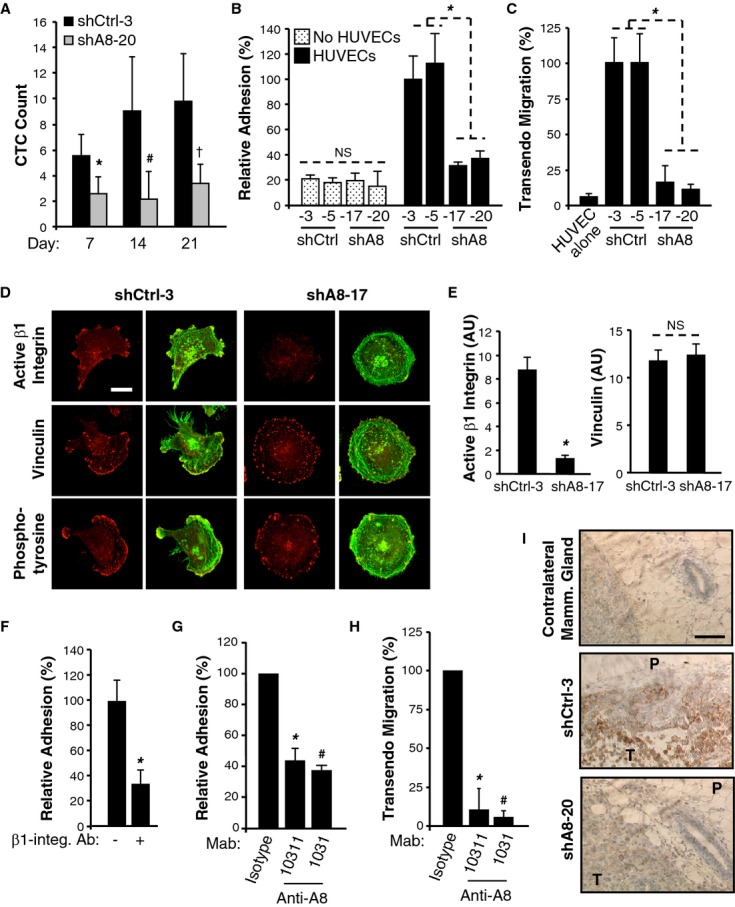
A Blood was drawn using the submandibular collection method from 4 mice/group on the indicated days after tumor cell implantation into the MFP and subjected to flow cytometry to measure GFP-positive CTCs. Average count for the 4 mice ± s.d. per μl of blood is given for each time point. * *P *= **0.03, ^#^ *P *= **0.05, ^†^ *P *= **0.02, Student's *t*-test. B Cells were incubated, in duplicate, on a HUVEC monolayer or in empty wells. Attached cells were counted in three fields per well. Mean ± s.d. from three independent experiments. NS, non significant, * *P *= **1.6E-6, Student's *t*-test. C Cells were subjected to an overnight transendothelial migration assay on HUVECs. Mean ± s.d. from three independent experiments. * *P *= **1.7E-5, Student's *t*-test. D, E Cells were plated on fibronectin-coated coverslips, stained with antibodies against active β1-integrin, vinculin or phosphotyrosine (focal adhesions markers) and phalloidin-488 (F-actin). Confocal microscopy images show adhesion protein channel (red) or merge with F-actin (green). Bar: 10 μm (D). Signal intensity for active β1-integrin and vinculin was determined using ImageJ software (arbitrary units). Mean intensity ± s.d. from >20 cells over 2 experiments. * *P *= **1.4E-4, NS, non significant, Student's *t*-test (E). F Adhesion of shCtrl-3 cells on HUVECs was assessed as in (B) with prior incubation of shCtrl-3 cells with an antagonist β1-integrin antibody (+) or a control isotype (−). Mean ± s.d. from three independent experiments. * *P *= **3.0E-5, Student's *t*-test. G, H Adhesion (G) and transendothelial migration (H) of shCtrl-3 cells on HUVECs was assessed as in B and C, respectively. Prior to the assays, shCtrl-3 cells were incubated either with monoclonal antibodies targeting the ectodomain of ADAM8 (Mab10311 or Mab1031) or an appropriate isotype control (IgG1 or IgG2B, respectively). The transmigration assay was performed for 9 h. Mean ± s.d. from three independent experiments. * *P *= **0.001, ^#^ *P *= **5.0E-4, Student's *t*-test (G). * *P *= **6.3E-7, ^#^ *P *= **4.0E-7, Student's *t*-test (H). I Active β1-integrin expression was assessed by immunohistochemistry in mouse mammary tumors (shCtrl-3 and shA8-20) and contralateral mammary glands (shCtrl-3) ( *n* = 7/group). P: Peritumoral area, T: Tumor. Bar: 100 μm.

Recent studies have demonstrated CTCs can be measured prior to formation of a detectable primary tumor (Eyles *et al*, 2010). Thus, we investigated whether the appearance of these early CTCs requires ADAM8 using a second set of mice ( *n* = 4/group). Blood was collected at day 7, when tumors were barely detectable and at days 14 and 21, when a difference in size was only beginning to be measured (Fig 4E). CTCs were detected as early as 7 days after cell implantation (Fig 6A). The numbers of CTCs were significantly lower in the mice injected with shA8-20 versus shCtrl-3 cells throughout the time course (Fig 6A). This suggests a lower potential risk for developing metastases to distant organs when ADAM8 is knocked down, independent of tumor size.

Binding of primary tumor cells and then CTCs to the endothelium followed by their transmigration through the blood vessel wall (intravasation and extravasation, respectively) are critical steps in metastasis (Steeg, 2006; Scott *et al*, 2012). Thus, the effect of ADAM8 on MDA-MB-231 cell binding to a layer of HUVECs was determined. shCtrl and shA8 cells were added to tissue culture plates in the presence or absence of a HUVEC monolayer. The two shCtrl clones were found to more efficiently adhere to a confluent HUVEC monolayer than the two shA8 clones, while adhesion on plastic was not affected by ADAM8 expression (Fig 6B). Furthermore, in a transendothelial cell migration assay, shA8 cells were much less effective in migrating through a confluent HUVEC monolayer than shCtrl cells (Fig 6C). Similar data were obtained with two ADAM8 siRNAs (supplementary Fig S7). Thus, ADAM8 promotes tumor cell adhesion onto and migration through an endothelial cell layer, as found in a vessel wall.

These findings suggest that ADAM8 participates in the establishment of cell-cell contact between breast tumor cells and endothelial cells. HUVECs do not express ADAM8 under normal conditions of culture (supplementary Fig S8), which excludes the possibility that ADAM8 forms dimers between tumoral and endothelial cells. Beta1-integrin has been shown to be involved in adhesion of MDA-MB-231 cells onto an endothelial cell monolayer (Price *et al*, 1996). Interestingly, using an antibody specific for the activated form of β1-integrin (12G10), we observed by immunofluorescence that shA8-17 cells displayed impaired β1-integrin activation, in contrast to shCtrl-3 cells (Fig 6D). Indeed, in the absence of ADAM8, the level of β1-integrin activation dropped by more than 4-fold, in contrast to another marker of focal adhesion, vinculin (Fig 6E).

As expected, the activation of β1-integrin in shCtrl-3 cells was required for adhesion to HUVECs, as judged by reduced binding *in vitro* following addition of an antagonist β1-integrin antibody (Fig 6F). A monoclonal antibody directed against the metalloprotease and disintegrin domains of ADAM8 (Mab1031) inhibited the adhesion of shCtrl-3 cells to HUVECs, essentially to the same extent as the β1-integrin antagonist antibody (Fig 6G) and also prevented their ability to transmigrate through an endothelial cell monolayer (Fig 6H). Interestingly, another monoclonal ectodomain antibody (Mab10311), which targets the extracellular cysteine-rich and EGF-like domains of ADAM8, had similar effects (Fig 6G and H). Importantly, using the specific 12G10 antibody, strong β1-integrin activation was observed in the mammary tumors derived from shCtrl-3 cells, notably at the peritumoral area, but was absent in shA8-20 tumors and in the contralateral mammary glands of shCtrl mice (Fig 6I), consistent with the hypothesis that ADAM8 promotes tumor cell adhesion onto the endothelium and dissemination via β1-integrin activation *in vivo*.

### ADAM8 ectodomain antibody reduces tumor growth and dissemination

**Figure 7:**
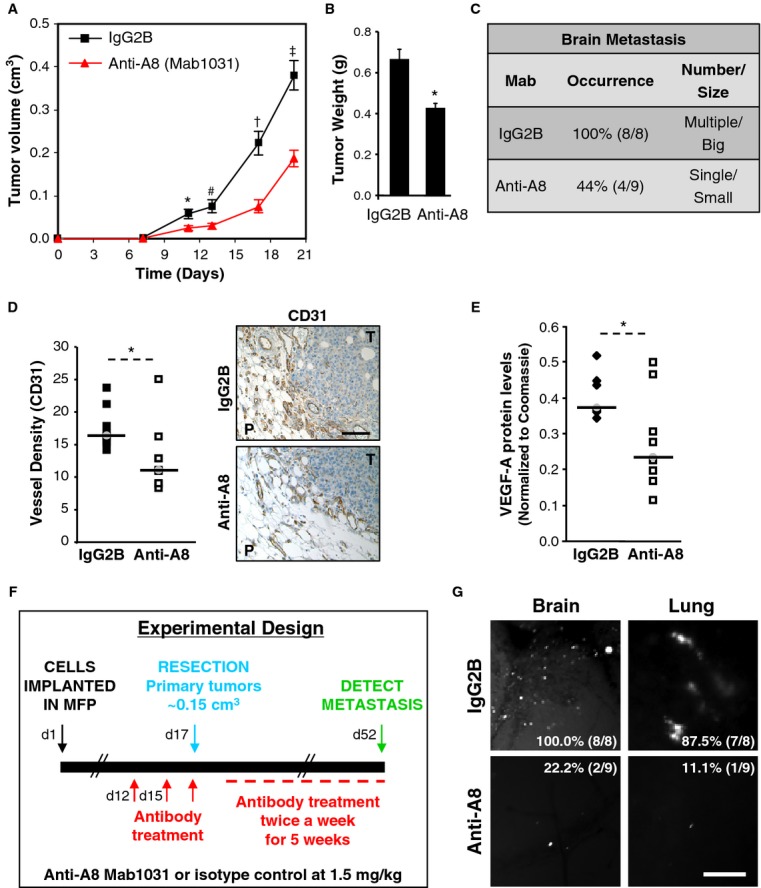
A–E MDA-MB-231 shCtrl-3 cells were injected into the MFP of female mice. Animals were treated with either 0.5 mg/kg anti-ADAM8 (anti-A8, Mab1031, *n* = 9) or isotype-matched control (IgG2B, *n* = 8) in i.p. injection twice weekly. Tumor volume was measured on the indicated days (mean ± s.e.m.). * *P *= **0.02 (day 11), ^#^ *P *= **0.01 (day 13), ^†^ *P *= **2.1E-4 (day 17), ^‡^ *P *= **1.5E-4 (day 20), Student's *t*-test (A). At the end of the experiment, tumors were weighed (mean ± s.e.m.). * *P *= **5.8E-4, Student's *t*-test (B) and the presence of brain metastases was examined by fluorescent microscopy (C). Angiogenesis was evaluated by CD31 immunohistochemical staining of tumor sections. Vessel density for each mouse is given as the average number of vessels in 2 slides/tumor (5 peritumoral hot spots/slide) and representative pictures are shown (D). P: Peritumoral area, T: Tumor. Bar: 100 μm. VEGF-A levels in the tumor extracts were determined by WB and normalized to Coomassie staining (E). * *P *= **0.04 (D), * *P *= **0.03 (E), Student's *t*-test. F–G Scheme of experimental design (F). Metastases to the brain and lungs were examined by fluorescent microscopy. Representative images and frequency of metastases (percentage of animals positive per group: IgG2B, *n* = 8; anti-A8, *n* = 9) are presented (G). Bar: 250 μm.

To validate ADAM8 as a therapeutic target for TNBC, the Mab1031 ADAM8 antibody was selected for use in an orthotopic model. Mice were treated with either 0.5 mg/kg of Mab1031 antibody ( *n* = 9) or a control isotype-matched IgG2B ( *n* = 8) by intra-peritoneal injection twice weekly from the day of implantation of shCtrl-3 cells. After 3 weeks, anti-ADAM8 treatment had significantly decreased primary tumor burden by approximately 50% (Fig 7A) and tumor weight by approximately 36% (Fig 7B). In animals treated with anti-ADAM8, a substantial reduction was also seen in the size and numbers of brain metastases per mouse as well as in the overall frequency (Fig 7C). Surrounding angiogenesis (Fig 7D) and VEGF-A levels in the tumors (Fig 7E) were similarly reduced by the ADAM8 antibody. Using the well-established ADAM8 metalloproteinase substrate CD23 (Koller *et al*, 2009) in an *in vitro* analysis, a substantial reduction in ADAM8 activity was observed upon addition of the anti-ADAM8 antibody (supplementary Fig S9).

To test the use of this ADAM8 antibody in a more clinically relevant setting, a tumor resection model was used (Fig 7F). Twelve days after mice were injected in the MFP with MDA-MB-231 shCtrl-3 cells, when tumors became palpable, treatment was initiated with either 1.5 mg/kg ADAM8 antibody or isotype control IgG2B. Antibody was further administered on day 15 and then on day 17 when tumors reached a volume of approximately 0.15 cm^3^ and tumor resection was performed. Antibody treatment was continued twice weekly for a 5-week period, and then organ metastasis was examined using fluorescent microscopy (Fig 7G). In the control group, metastases to the brain and lungs were seen in 8/8 and 7/8 mice, respectively; whereas in the anti-ADAM8-treated group only 2/9 and 1/9 mice showed metastases in these organs. Thus, treatment with an anti-ADAM8 antibody resulted in a profound reduction in the frequency of metastases to both the brain and lungs compared to the isotype control antibody.

## Discussion

**Figure 8:**
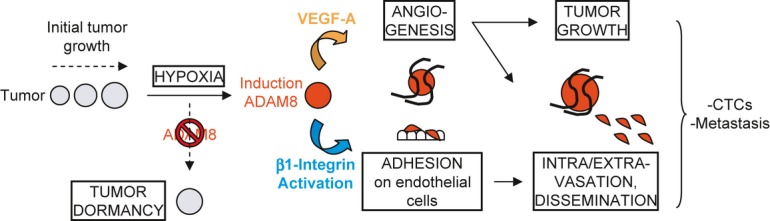
When solid tumors reach a few millimeters in diameter, hypoxic stress is induced, which leads to ADAM8 induction resulting in strong pro-angiogenic signaling, in part via VEGF-A release into the extracellular compartment, and endothelial cell recruitment. ADAM8 also promotes β1-integrin activation on tumor cells needed for their adhesion onto and transmigration through the blood vessel wall, which supports dissemination of CTCs and development of metastases. Importantly, here we demonstrate that if the induction of ADAM8 is blocked or its activity inhibited with antibody, there is an insufficient angiogenic response, leading to tumor mass dormancy or slowing of growth, as well as to a striking reduction of CTCs and metastases.

Overall, our findings validate ADAM8 as a therapeutic target for TNBC. ADAM8, which is abundantly expressed in aggressive human breast tumors and their metastases, was shown to be essential for tumor growth and dissemination in orthotopic mouse models using knockdown and antibody strategies. Notably, treatment of pre-existing tumors with an antibody targeting ADAM8 profoundly reduced their ability to metastasize in a resection model. Two essential novel roles for ADAM8 in tumor progression were identified (Fig 8). ADAM8 facilitates the release of pro-angiogenic factors, including VEGF-A, into the tumor microenvironment, leading to neovascularization and continued tumor growth. ADAM8 also activates β1-integrin on the tumor cells permitting their attachment to the vascular endothelium and entry into the blood circulation. Increased numbers of CTCs raise the risk of developing metastases in distant organs such as brain and lungs. While TNBCs are highly aggressive and more likely to spread compared to other breast tumors, no targeted treatment options are currently available. Given the importance of ADAM8 in growth and dissemination of TNBC, and its advantageous localization on the tumor cell surface, our studies identify ADAM8 as a promising novel druggable target for the treatment of these breast cancers.

Binding of integrins to cell-surface adhesion molecules and extracellular matrix proteins governs cell adhesion and shape via transduction of intracellular signals (Avraamides *et al*, 2008; Desgrosellier & Cheresh, 2010). Beta1-integrin was found to mediate adhesion of breast cancer and other tumor cells to quiescent endothelial cells (Price *et al*, 1996). Several ADAM proteins, which contain RGD-like integrin-binding motifs in their disintegrin domain, constitute ligands for integrins (Bridges & Bowditch, 2005). Bartsch and coworkers recently observed that ADAM8 is also required for activation of β1-integrin in pancreatic cancer cells (Schlomann *et al*, in revision). Furthermore, using a novel cyclic peptide inhibitor that binds to the ADAM8 disintegrin domain, they demonstrated a profound reduction in tumor burden and metastasis in orthotopic xenograft and *Kras*^*G12D*^ transgenic mouse models of pancreatic cancer. Similarly, an antibody targeting the metalloproteinase/disintegrin domains of ADAM8 greatly reduced the ability of TNBC cells to bind endothelial cells *in vitro* and to disseminate in mice. In addition, another antibody directed against the ADAM8 Cys-Rich/EGF-like domains was equally effective *in vitro*, suggesting allosteric hindrance or altered ADAM8 structure may also play a role.

Early in the tumorigenesis process, solid tumors stop increasing in mass (tumor dormancy) unless they can overcome hypoxic stress by stimulating angiogenesis, which provides oxygen and nutrients essential for further tumor growth (Bergers & Benjamin, 2003; Naumov *et al*, 2006; Majmundar *et al*, 2010). In TNBC cells, hypoxia induced expression of ADAM8 proform and active processed form. *In vivo*, mammary tumors of shCtrl, but not shA8 animals, displayed a substantial increase in ADAM8 protein around necrotic areas. Using Transfac analysis, several putative Hypoxia Response Elements were identified in the *ADAM8* promoter, although their functional roles have not as yet been tested. Of note, the extent of HIF-1α induction in shCtrl versus shA8 cells and of necrosis in tumors derived from these cells was similar, suggesting ADAM8 KD does not alter the initial response to hypoxia. High ADAM8 levels were also seen in patient TNBCs, especially in the metastases to the brain, consistent with induction related to hypoxic stress. Altogether, these findings are consistent with the hypothesis that induction of ADAM8 is required for breast tumors to overcome hypoxic stress.

Activation of the VEGFR2 tyrosine kinase receptor by VEGF-A, which promotes endothelial cell proliferation, sprouting, migration, as well as vascular permeability, is a major pathway inducing angiogenesis (Lohela *et al*, 2009). High VEGF-A expression has been associated with increased microvessel density, metastasis, and a shorter overall survival in patients with primary invasive breast cancer (Mohammed *et al*, 2007). VEGF-A activity is regulated to a large extent in the extracellular space, where cell-surface or extracellular matrix bound VEGF-A reservoirs can be made bioavailable through proteolytic cleavage by various enzymes, including metalloproteinases MMP-3,-7 and-9 (Lee *et al*, 2005; Hawinkels *et al*, 2008). TNBCs with higher intratumoral VEGF-A levels compared to non-TNBCs have a shorter time to relapse (Linderholm *et al*, 2009). KD of ADAM8 in MDA-MB-231 cells resulted in reduced VEGF-A levels in conditioned media, but did not appear to affect *VEGF-A* mRNA levels (supplementary Fig S10). Consistently, both ADAM8 antibody treatment and KD reduced tumor angiogenesis. As a positive correlation was identified between *ADAM8* and *CD31* mRNA levels in basal-like tumors, high ADAM8 levels appear associated with increased tumor vascularization in these patients. Our data suggest that the metalloproteinase activity of ADAM8 plays an essential role in processing or release of VEGF-A and possibly other pro-angiogenic factors.

Important biological roles of ADAM proteins in malignancy have been previously identified (Duffy *et al*, 2009). ADAM17 promotes epithelial and endothelial cell migration, and vasculogenesis (Duffy *et al*, 2011; Maretzky *et al*, 2011). ADAM15 cytoplasmic domain variants have been implicated in mammary carcinoma (Zhong *et al*, 2008), while an ADAM12 secreted isoform promotes breast tumor metastasis *in vivo* (Roy *et al*, 2011a). ADAM10 was found upregulated in melanoma metastasis compared to primary melanoma (Lee *et al*, 2010). Previously, ADAM8 expression was correlated with tumor stage or an unfavorable prognosis for patients with esophageal-gastric, lung, pancreatic or brain cancers, and with invasiveness of tumor cells in culture (Koller *et al*, 2009; Baren *et al*, 2012), although little was explored about its mechanism of action. Whereas there is no evidence of *ADAM8* gene amplification in many of these cancers, several pathways commonly dysregulated in aggressive malignancies were found to induce *ADAM8* mRNA levels, e.g., RelB NF-κB and EGFR (Sriraman *et al*, 2008).

In the present study, ADAM8 expression levels correlated with a poor prognosis in breast cancer patients and concomitantly with increased numbers of CTCs and metastases in an orthotopic mouse model. It has been recently shown that CTCs and micrometastases occur early during the progression of cancers from various origins (melanoma, breast and cervical cancers) (Toh *et al*, 2012), suggesting that therapies neutralizing the adhesion of these CTCs onto endothelium could be extremely beneficial. Furthermore, ADAM8 protein levels were found to be higher in the serum of patients with breast cancer versus benign breast disease. The combination of ADAM8 and ADAM12 activities measured in urine samples was shown to identify with 90% confidence breast cancer patients with invasive and metastatic disease (Roy *et al*, 2011b). These findings suggest that further investigation into the potential use of monitoring ADAM8 in a non-invasive method as a diagnostic/prognostic biomarker for early cancer detection or potentially to follow disease progression and recurrence is warranted.

## Materials and Methods

### Cell treatment conditions

For culture in 3D suspension conditions, single-cell suspensions in complete media were plated at 1.25 × 10^4^ cells per well in ultra low-attachment 6-well plates (Costar) and incubated for 48 h. For culture in hypoxic conditions, cells were incubated in 1% oxygen for 6 or 24 h in 6-well plates.

### siRNA and shRNA knockdown analyses

Transient RNAi-mediated ADAM8 KD was performed with the following short interfering RNAs (siRNAs) (QIAGEN, Valencia, CA, USA):

siADAM8 (siA8)#1 (Hs_ADAM8_6): 5′-CGGCACCTGCATGACAACGTA-3′

siA8#2 (Hs_ADAM8_7): 5′-CTGCGCGAAGCTGCTGACTGA-3′

AllStar negative control siRNA (QIAGEN) was used in each experiment as a non-silencing control siRNA (siCtrl). siRNAs (10 nM) were introduced in cells using Lipofectamine RNAi Max Transfection Reagent (Invitrogen, Carlsbad, CA, USA) by reverse transfection according to the manufacturer's protocol. Transfected cells were used 24 h later in functional assays.

The target sequence of the *ADAM8* shRNA used in this study was GCGGCACCTGCATGACAACGTACAGCTCA.

### Endothelial cell adhesion assay

Endothelial cell adhesion assays were performed as previously described (Price *et al*, 1996). Briefly, 1 × 10^5^ HUVEC cells were plated, in duplicate, in 48-well plates. After 24 h, 5 × 10^4^ MDA-MB-231 cells stained with NucBlue (Invitrogen) were incubated for 15 min at 37°C in 300 μl of EBM-2 Basal Medium supplemented with 1% FBS on top of the confluent HUVEC monolayer or in empty wells. Unattached MDA-MB-231 cells were washed three times with PBS and attached NucBlue-positive cells were counted in three random fields/well ( *n* = 6). The average percentage relative to control samples is presented ± s.d. from three independent experiments. For inhibition experiments with antibodies, tumor cells were pre-treated with 10 μg/ml of β1-integrin antibody (BD Biosciences, 552828), 20 μg/ml of ectodomain ADAM8 antibody (Mab1031 and Mab10311; R&D Systems, Minneapolis, MN, USA), or control isotypes (rat or mouse, respectively) for 30 min at room temperature and then washed twice with PBS before being used.

### Human Angiogenesis array

Levels of 55 angiogenesis related proteins secreted into the medium of shCtrl and shA8 MDA-MB-231 clones over a 24 h period was assessed using the Proteome Profiler™ Human Angiogenesis antibody array (R&D Systems, ARY007). Briefly, 2% FBS conditioned medium was centrifuged at 3000 *g*. for 10 min to remove cell debris and a volume of medium corresponding to 2.5 × 10^5^ cells was subjected to the antibody array according to the manufacturer's protocol. The signal intensity of the detected proteins (two spots for each protein) was measured using ImageJ (NIH, Bethesda, MD, USA), the background signal subtracted and the average intensity (Mean ± s.d.) for the two clones calculated.

### Immunofluorescence microscopy

Cells were plated on fibronectin-coated coverslips, fixed and stained with antibodies against active β1-integrin (12G10; Millipore, Temecula, CA, USA), vinculin or phosphotyrosine, and phalloidin-488 (F-actin). A mouse Alexa-568 was used as secondary antibody (Invitrogen). Cells were analyzed by Confocal microscopy using a Nikon A1R microscope equipped with a CFI Plan Fluor 40× oil-objective. Images, which were captured with NIS Element software (Nikon, Melville, NY, USA), show adhesion protein channel (red) or merge with F-actin (red/green overlay). Quantification of intensity of signal was determined using ImageJ software (arbitrary unit) and mean ± s.d. from >20 cells over two independent experiments is given.

### MFP mouse model

Six-week-old female nonobese diabetic/severe combined immunodeficient (NOD/SCID) mice (Jackson Laboratory, Bar Harbor, ME, USA) were implanted with 2.5 × 10^6^ cells in 50 μl of a 1:1 dilution of Matrigel (CB-40230A; BD Biosciences, San Jose, CA, USA) and DMEM medium, in the fourth inguinal MFP. Primary tumor growth was monitored by caliper measurement twice a week. Tumor volumes were calculated as (length × width^2^)/2. Mice were sacrificed when tumors derived from shCtrl-3 MDA-MB-231 cells had reached a volume of approximately 1 cm^3^. Mice were photographed, tumors were dissected, photographed, weighed and fixed in 10% Neutral Buffered Formalin for histological and IHC analyses. For antibody treatment experiments, control isotype IgG2B ( *n* = 8) or anti-A8 mAb ( *n* = 9) (Mab004 and Mab1031 respectively; R&D Systems) was injected i.p. twice weekly at 0.5 mg/kg from the day of shCtrl-3 cell implantation in the MFP. Alternatively, treatment with doses of 1.5 mg/kg was performed after the primary tumors became palpable and continued with an injection on the day of resection when the tumors reached a volume of approximately 0.15 cm^3^ and twice weekly for the following 5 weeks. For detection of metastasis, brains and lungs were dissected and examined for GFP expression using a fluorescent dissecting microscope (Leica MZ FLIII, Buffalo Grove, IL, USA).

### Detection of CTCs

Blood was either collected at sacrifice (day 33) by cardiac puncture (˜500 μl) or drawn from mice using the submandibular blood collection method (˜100 μl) 7, 14 and 21 days after inoculation of tumor cells into the MFP. In order to evaluate level of autofluorescence, blood was also collected from NOD/SCID mice that were not injected with any tumor cells. All blood samples were collected in heparin (100 USP units/sample), diluted and processed the same day for detection of GFP-positive CTCs using a confocal flow cytometry system at a flow rate of 4.5 μl/min, as previously described (Greiner *et al*, 2011). Enumerated cells were identified with a previously described algorithm based on the combined detection of peaks appearing simultaneously in a fluorescence channel optimized for GFP detection and in at least one of the channels detecting scattering at either 405 or 488 nm (Hwu *et al*, 2011) (see supplementary materials).

### ELISA analysis of breast tissue and serum

Following surgical resection, tissues were snap-frozen in liquid nitrogen and homogenized using a Mikro-Dismembrator (Braun Biotech International, Melsungen, Germany). Supplementary Table S1 summarizes the characteristics of the primary breast carcinomas analyzed. The normal breast tissues used were samples adjacent to carcinomas. In addition, fibroadenomas were studied. Protein was extracted from tissue samples using 50 mM Tris–HCl (pH 7.4) containing protease inhibitors (Roche, Dublin, Ireland) and 1% Triton X-100 under agitation at 4°C for 1 h. Blood samples were collected in BD Vacutainer red-top tubes (BD Biosciences, 367837) from consenting women who attended the Breast Clinic at St Vincent's University Hospital (SVUH, Dublin, Ireland) as previously described (Pierce *et al*, 2010), and had been referred to the hospital with the presence of a breast lesion or lump. At time of sampling, it was unknown whether the patient had benign or malignant breast disease. Blood samples were allowed to clot for 1 h and centrifuged at 400 *g* for 10 min. Sera were stored at −80°C within 2 h of being taken. Detailed clinicopathological characteristics of the cancers diagnosed are listed in supplementary Table S2. ADAM8 protein was measured in serum and tumor extracts using an ELISA assay (R&D Systems) according to the manufacturer's instructions.

### Immunohistochemistry of patient samples

Paraffin-embedded tissues from Formalin-fixed tumors were selected (see supplementary Table S3). Sections of 3-μm-thickness were deparaffinized in xylene and rehydrated through an alcohol gradient, and endogenous peroxidase activity was blocked with 3% hydrogen peroxide. Samples were steamed before incubation for antigen retrieval with citrate buffer (pH 6.0). Slides were incubated with a primary antibody targeting ADAM8 (1:150, B4068, LSBio) on an automated immunostainer using a labelled streptavidin-biotin method (Dako, Trappes, France) followed by 3,3′-diaminobenzidine chromogen detection. Immunostained slides were counterstained with hematoxylin. Negative controls were included in each run by omitting the primary antibody. ADAM8 immunoreactivity in carcinomatous cells was scored by a pathologist using a 5-tiered semiquantitative system (0: no staining, 1+: focal weak staining <10%, 2+: 10–25%, 3+: 25–50%, 4+: >50%). Non-carcinomatous cells were excluded from scoring.

### Immunohistochemistry of mouse samples

Primary antibodies targeting ADAM8 (1:150, B4068; LSBio, Seattle, WA, USA), CD31 (1:50, ab28364; Abcam, Cambridge, MA, USA) and the active conformation of β1-integrin (1:100, B2861; LSBio) were used in the same protocol described as above for patient specimens. For evaluation of vessel density, 2 slides/tumor were cut 200 μm apart and subjected to immunostaining for CD31. Three (ADAM8 KD experiment) or five (anti-ADAM8 experiment) areas with the greatest numbers of vessels (hot spots) at the tumor periphery were identified at low magnification for each tumor slice and photographed at 20× magnification. The numbers of vessels/hotspot were counted and the average vessel density/tumor plotted ( *n* = 6/tumor).

### Gene expression and statistical analysis

Cancer datasets were downloaded from Oncomine (Compendia Bioscience, http://www.oncomine.com), as previously described (Romagnoli *et al*, 2012). The rank for *ADAM8* across the 14 analysis and its *P*-value for the comparison of *ADAM8* expression between breast carcinoma and normal breast, were obtained directly from the Oncomine website. A comprehensive gene expression microarray data set of 295 primary breast tumors (obtained from Rosetta Inpharmatics) prepared by van de Vijver and colleagues (van de Vijver *et al*, 2002), was used to compare *ADAM8* mRNA levels across the different molecular breast cancer subtypes and to assess its correlation with patient outcome. Univariate relationships between *ADAM8* mRNA log ratio data and patient outcome were assessed using the Log-rank test. A relative risk (RR) of more than 1 indicates an increased risk of relapse or mortality. Confidence intervals (CI) were calculated for the RR, which was then tested for statistical significance using the Wald test. Data were analyzed using PASW statistics version 18.0 (SPSS Inc., Chicago, IL, USA), which provides exact *P*-values when above 0.0001. Results are presented using Kaplan–Meier curves. The Pearson's pairwise correlation plot showing a correlation between *ADAM8* and *CD31* ( *PECAM1*) expression and its *P*-value were obtained directly from the bc-GenExMiner microarray database (http://bcgenex.centregauducheau.fr). All other analyses were performed using a two-tailed Student's *t*-test for samples with equal variance. *P-*value < 0.05 was considered statistically significant.

### Study approval

All animal work was done in accordance with a protocol approved by the Institutional Animal Care and Use Committee of Tufts University and Tufts Medical Center. Patient samples were obtained with institutional board approval from St. Vincent's University Hospital (Dublin, Ireland). Informed consent was obtained from patients to use their surgical specimen and clinicopathological data for research purposes according to the French Committee for the protection of Human Subjects.

**The paper explained**

**Problem**

Despite recent advances in breast cancer treatment, over 500 000 women die yearly worldwide. Triple-negative breast cancers (TNBCs) represent only 15–20% of all breast cancer cases, but are highly aggressive and lack targeted therapies due to the absence of steroid hormone and HER2 receptors. TNBC frequently recurs, responds poorly to chemotherapy and accounts for more than 25% of breast cancer deaths. Thus, there is an urgent need for new targets for the treatment of TNBC. Here, we validate the transmembrane metalloprotease-disintegrin protein ADAM8, which is non-essential under physiological conditions, as a target for TNBC treatment.

**Results**

ADAM8 was found abundantly expressed in breast cancer, especially in TNBC compared to normal tissue, and its expression correlated with poor patient outcome. Consistently, ADAM8 promoted the aggressive phenotype of TNBC cells in culture. In a mouse orthotopic model, TNBC cells with ADAM8 knockdown failed to grow beyond a palpable size due to reduced angiogenesis. Circulating tumor cells in the blood and brain metastases were also significantly decreased. In breast cancer patients, ADAM8 was detected in approximately 50% of metastases. Two critical functions of ADAM8 were demonstrated: (i) stimulation of angiogenesis via release of VEGF-A and other pro-angiogenic factors; (ii) activation of β1-integrin on the surface of TNBC cells required for binding to the endothelium and subsequent extra/intravasation. Anti-ADAM8 ectodomain antibody treatment initiated at the time of TNBC cell implantation greatly reduced primary tumor burden and metastasis in an orthotopic model. Furthermore, antibody treatment of established tumors profoundly decreased metastases in a resection model.

**Impact**

This study identifies ADAM8 as a crucial player in multiple steps of breast tumorigenesis and metastatic spread, notably in angiogenesis and cancer cell adhesion to the endothelium. The results validate the transmembrane ADAM8 protein as a promising novel target for the treatment of aggressive TNBC and metastatic breast disease, and suggest ADAM8 is accessible to antibody-based therapeutic approaches.

Supporting information is also available, including cell lines and culture conditions, western blotting, and assays of ATP levels, growth in soft agar, Matrigel outgrowth, migration, invasion, tube formation, transendothelial migration, ADAM8 protease activity, and CTC detection.
