# Supplementary material for: ADAM8 expression in invasive breast cancer promotes tumor dissemination and metastasis
Source: EMBO Mol Med. 2013 Dec 27;6(2):278–94. doi: 10.1002/emmm.201303373 (PMC3927960; doi:10.1002/emmm.201303373)
Supplement: Supplementary file 1 [file emmm0006-0278-sd1.pdf]

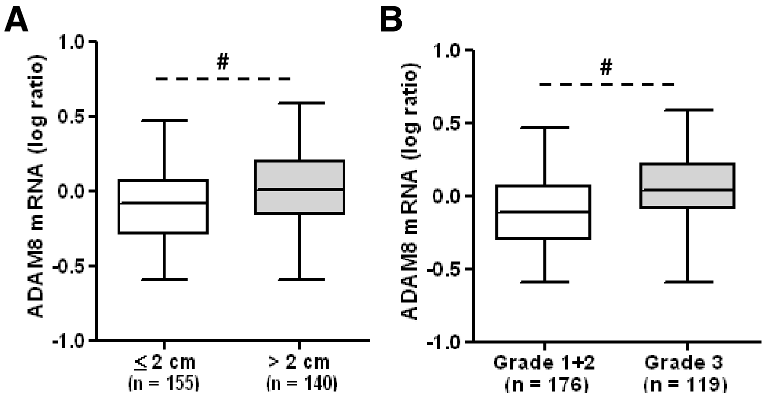

Supplementary Fig S1. *ADAM8* mRNA levels correlate with clinicopathological variables of human breast tumors.

(A-B) The van de Vijver microarray dataset (van de Vijver et al, 2002) was used to compare *ADAM8* mRNA levels and clinicopathological features of 295 human breast tumors: size (A) and grade (B). $^{\#}P$ = 0.001, Mann-Whitney *U* test.
