# Supplementary material for: ADAM8 expression in invasive breast cancer promotes tumor dissemination and metastasis
Source: EMBO Mol Med. 2013 Dec 27;6(2):278–94. doi: 10.1002/emmm.201303373 (PMC3927960; doi:10.1002/emmm.201303373)
Supplement: Supplementary file 2 [file emmm0006-0278-sd2.pdf]

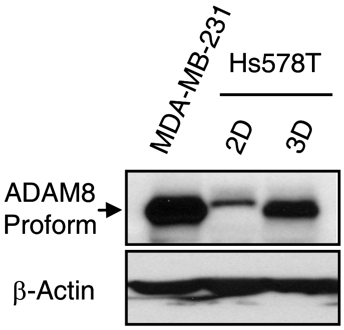

Supplementary Fig S2. ADAM8 proform is induced in Hs578T cells grown in 3D-cultures, which co-migrates with the one seen in MDA-MB-231 cells.

Hs578T cells were cultured in adherent conditions (2D) or in suspension on ultra low-attachment plates (3D) for 48 h. MDA-MB-231 cells were cultured in adherent conditions only. Whole-cell extracts (WCEs) were analyzed by Western blotting for ADAM8 (LSBio antibody) and for β-Actin.
