# Supplementary material for: ADAM8 expression in invasive breast cancer promotes tumor dissemination and metastasis
Source: EMBO Mol Med. 2013 Dec 27;6(2):278–94. doi: 10.1002/emmm.201303373 (PMC3927960; doi:10.1002/emmm.201303373)
Supplement: Supplementary file 3 [file emmm0006-0278-sd3.pdf]

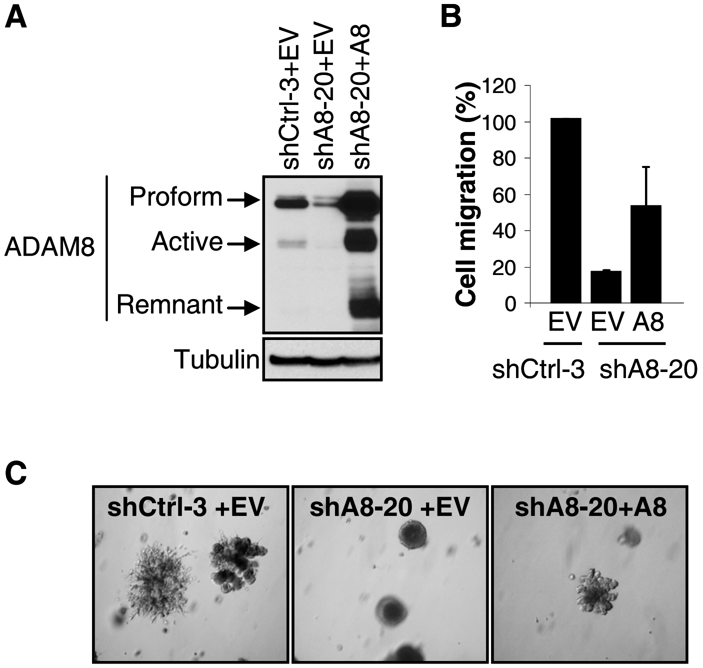

Supplementary Fig S3. Ectopic ADAM8 expression rescues the invasive phenotype of stable ADAM8 knockdown cells.

ADAM8 (A8) or empty vector (EV) DNA was transiently transfected into shA8-20 MDA-MB-231 cells for 24 h.

(A) WCEs were isolated and subjected to Western blot analysis for ADAM8 (LSBio antibody) and Tubulin.

(B-C) Migration and Matrigel outgrowth assays were performed as described in Figures 3C and 3D, respectively. Ectopic expression of ADAM8 in shA8-20 cells partially rescued their ability to migrate (B) and to form invasive colonies (C), consistent with the observed transfection efficiency of ~40%.
