# Supplementary material for: ADAM8 expression in invasive breast cancer promotes tumor dissemination and metastasis
Source: EMBO Mol Med. 2013 Dec 27;6(2):278–94. doi: 10.1002/emmm.201303373 (PMC3927960; doi:10.1002/emmm.201303373)
Supplement: Supplementary file 4 [file emmm0006-0278-sd4.pdf]

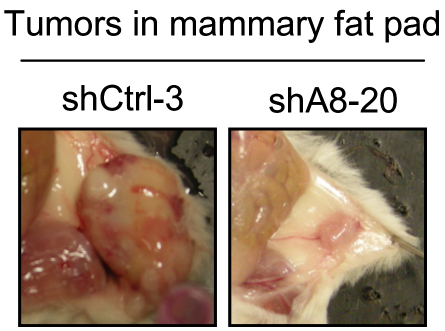

Supplemental Fig S4. ADAM8 knockdown tumors fail to grow beyond a palpable size and are poorly vascularized in a mammary fat pad mouse model.

MDA-MB-231-derived shCtrl-3 and shA8-20 cells were injected into the mammary fat pad of mice (n = 7/group). At the end of the experiment, tumors were photographed *in situ*. Representative photographs displaying the decreased size and poor vascularization of the shA8-20 vs shCtrl-3 tumors are shown.
