# Supplementary material for: ADAM8 expression in invasive breast cancer promotes tumor dissemination and metastasis
Source: EMBO Mol Med. 2013 Dec 27;6(2):278–94. doi: 10.1002/emmm.201303373 (PMC3927960; doi:10.1002/emmm.201303373)
Supplement: Supplementary file 5 [file emmm0006-0278-sd5.pdf]

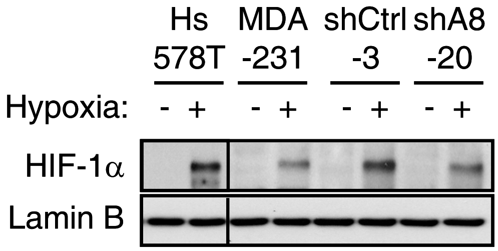

Supplemental Fig S5. HIF-1α is induced in breast cancer cell lines under hypoxia. Cells were incubated for 8 h under hypoxic conditions and nuclear extracts were subjected to Western blotting analysis for HIF-1α (Novus Biologicals, NB100-105) and Lamin B (Santa Cruz Biotechnology, sc-6217). All lanes were from the same gel, but two different times of exposure were used for the Hs578T cells (5 min) vs the MDA-MB-231-derived lines (30 min), as indicated by the vertical line.
