# Supplementary material for: ADAM8 expression in invasive breast cancer promotes tumor dissemination and metastasis
Source: EMBO Mol Med. 2013 Dec 27;6(2):278–94. doi: 10.1002/emmm.201303373 (PMC3927960; doi:10.1002/emmm.201303373)
Supplement: Supplementary file 6 [file emmm0006-0278-sd6.pdf]

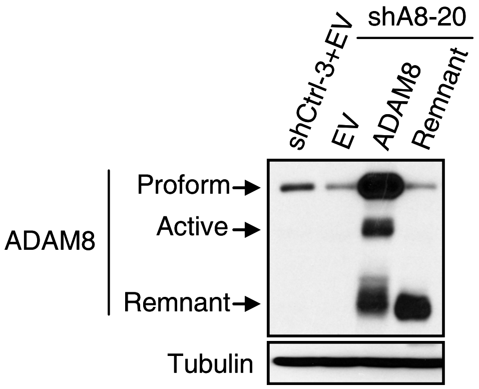

Supplementary Fig S6: Ectopic expression of full-length ADAM8 and remnant form in stable ADAM8 knockdown cells.

MDA-MB-231-derived shCtrl-3 or shA8-20 clones were transfected with the indicated vectors expressing ADAM8, its remnant form (Remnant) or empty vector (EV) DNA for 24 h. Then, the medium was replaced by serum-free medium and incubated for 16 h. Supernatants and cells were collected. Cells were lysed and WCEs were assessed by Western blotting for ADAM8 expression. In parallel, VEGF-A released into the media was measured (presented in Figure 5I). The data indicated that the remnant form was unable to induce the release of VEGF-A from shA8-20 cells, in contrast to the full-length ADAM8.
