# Supplementary material for: ADAM8 expression in invasive breast cancer promotes tumor dissemination and metastasis
Source: EMBO Mol Med. 2013 Dec 27;6(2):278–94. doi: 10.1002/emmm.201303373 (PMC3927960; doi:10.1002/emmm.201303373)
Supplement: Supplementary file 7 [file emmm0006-0278-sd7.pdf]

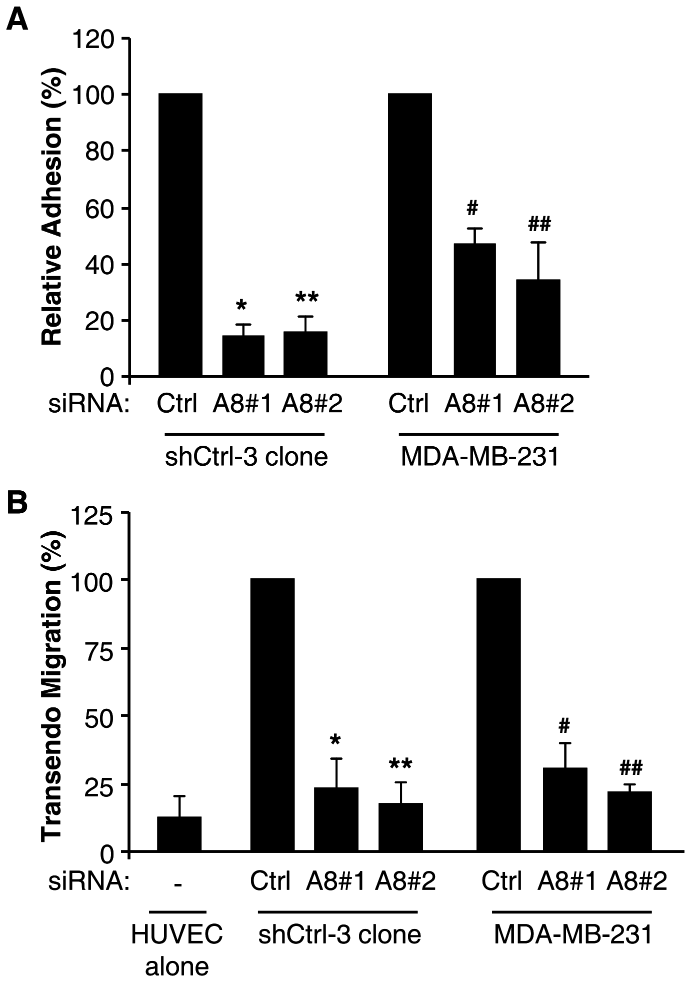

Supplementary Fig S7. siRNA-mediated knockdown of ADAM8 in MDA-MB-231 cells reduces their ability to adhere to and transmigrate through HUVECs.

(A-B) The MDA-MB-231-derived shCtrl-3 clone and the total MDA-MB-231 population were transfected with either *Control* siRNA (siCtrl) or two specific *ADAM8* siRNAs (siA8) for 72 h. Cancer cell adhesion to (A) and transendothelial migration through (B) HUVECs were assessed as in Figure 6B and 6C, respectively. **P* = 0.001, ***P* = 0.001, $^{\#}P$ = 0.005, $^{\#\#}P$ = 0.005, Student's *t* test, n = 2 (A). **P* = 0.008, ***P* = 5.9E-4, $^{\#}P$ = 0.009, $^{\#\#}P$ = 6.6E-4, Student's *t* test, n = 2 (B).
