# Supplementary material for: ADAM8 expression in invasive breast cancer promotes tumor dissemination and metastasis
Source: EMBO Mol Med. 2013 Dec 27;6(2):278–94. doi: 10.1002/emmm.201303373 (PMC3927960; doi:10.1002/emmm.201303373)
Supplement: Supplementary file 8 [file emmm0006-0278-sd8.pdf]

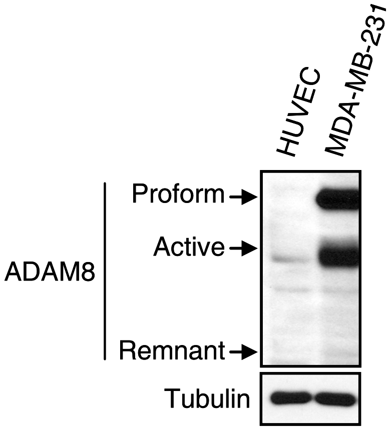

Supplemental Fig S8. ADAM8 is not detected in HUVECs.

WCEs from HUVECs and MDA-MB-231 cells, as a positive control for ADAM8 expression, were subjected to Western blotting for ADAM8 (LSBio antibody) and Tubulin.
