# Supplementary material for: ADAM8 expression in invasive breast cancer promotes tumor dissemination and metastasis
Source: EMBO Mol Med. 2013 Dec 27;6(2):278–94. doi: 10.1002/emmm.201303373 (PMC3927960; doi:10.1002/emmm.201303373)
Supplement: Supplementary file 9 [file emmm0006-0278-sd9.pdf]

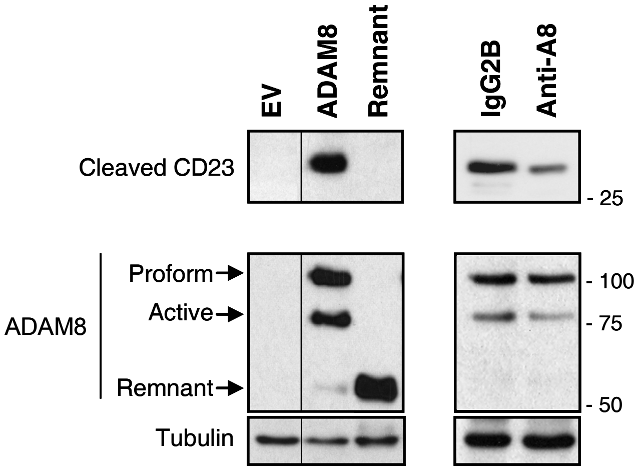

Supplemental Fig S9. Anti-ADAM8 antibody inhibits the metalloprotease activity of ADAM8.

(Left) HEK293 cells were co-transfected with vectors expressing CD23, which is a well-established substrate of ADAM8 protease activity, and either ADAM8 or its remnant form or EV DNA. Six hours later, the medium was replaced with serum-free medium. After 16 h, supernatants were collected and volumes corresponding to equal cell numbers were assessed for cleaved CD23 (29 kDa) using Western blotting. In parallel, cells were harvested and WCEs were assessed for expression of ADAM8 and Tubulin. All lanes were from the same gel, but cut to re-align as indicated by the vertical line. In the presence of full-length ADAM8 but not its remnant form lacking the metalloprotease domain, CD23 is cleaved with release of a diagnostic soluble 29 kDa fragment.

(Right) HEK293 cells were co-transfected with vectors expressing CD23 and ADAM8. Six hours later, medium was replaced with serum-free medium in the presence of 10 μg/ml of either anti-ADAM8 antibody (Mab1031) or isotype control IgG2B (Mab004). After 16 h, we measured cleaved 29 kDa CD23b in supernatants and ADAM8 expression in WCEs, as described above. In the presence of the anti-ADAM8 antibody, the cleavage of CD23 by ADAM8 was substantially reduced.
