# Supplementary material for: ADAM8 expression in invasive breast cancer promotes tumor dissemination and metastasis
Source: EMBO Mol Med. 2013 Dec 27;6(2):278–94. doi: 10.1002/emmm.201303373 (PMC3927960; doi:10.1002/emmm.201303373)
Supplement: Supplementary file 10 [file emmm0006-0278-sd10.pdf]

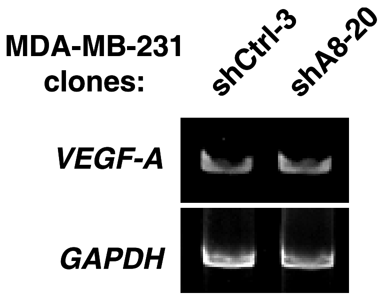

Supplementary Fig S10. ***VEGF-A*** **mRNA levels are unchanged in stable ADAM8 knockdown cells.**

RNA was isolated using NucleoSpin RNA II (Macherey-Nagel) according to the manufacturer's protocol and quantified by measuring the A_{260}. For reverse transcription-PCR (RT-PCR), 1 µg of RNA was reverse transcribed with SuperScript III reverse transcriptase in the presence of 250 ng of random primers (Invitrogen). PCR was performed in a thermal cycler with the following conditions and primer sets:

VEGF-A sense: 5'-GCAGAATCATCACGAAGTGG-3'

VEGF-A antisense: 5'-GCATGGTGATGTTGGACTCC-3'

95°C for 30 sec, 60°C for 30 sec and 72°C for 45 sec; 24 cycles

GAPDH sense: 5'-TCACCATCTTCCAGGAG-3'

GAPDH antisense: 5'-GCTTCACCACCTTCTTG-3'

95°C for 30 sec, 55°C for 30 sec and 72°C for 45 sec; 18 cycles
