# Supplementary material for: ADAM8 expression in invasive breast cancer promotes tumor dissemination and metastasis
Source: EMBO Mol Med. 2013 Dec 27;6(2):278–94. doi: 10.1002/emmm.201303373 (PMC3927960; doi:10.1002/emmm.201303373)
Supplement: Supplementary file 11 [file emmm0006-0278-sd11.pdf]

Supplementary Table S1. Relationship between *ADAM8* mRNA (continuous log ratio levels) and disease-free survival (top table) and overall survival (bottom table) in 295 breast cancer patients. Data was analysed using a multivariate Cox model. CI; Confidence Interval.

	**Wald test *P*-value**	**Relative Risk**	**95% CI**
Number of positive nodes	0.238	1.057	0.964 – 1.159
ER Category[1]	0.901	0.970	0.600 – 1.568
Size (≤ 2cm, > 2 cm)	0.044	1.537	1.011 – 2.336
Tumour Grade	0.004	1.575	1.152 – 2.154
HER-2 Status[2]	0.248	1.333	0.879 – 2.171
ADAM-8	**0.001**	4.470	1.877 – 10.644

	**Wald test *P*-value**	**Relative Risk**	**95% CI**
ER Category[1]	0.055	0.607	0.365 – 1.011
Tumour size (≤, > 2cm)	0.069	1.558	0.966 – 2.513
Grade Category	**0.002**	1.860	1.259 – 2.748
Age (yrs)	**0.047**	0.962	0.926 – 0.999
HER-2 Status[2]	0.132	1.498	0.886 – 2.535
ADAM-8	**0.052**	2.707	0.992 – 7.384

[1] Estrogen Receptor alpha expression, as measured by microarray (van de Vijver et al, 2002)
[2] HER2 status, as determined by microarray (van de Vijver et al, 2002)
