# Supplementary material for: ADAM8 expression in invasive breast cancer promotes tumor dissemination and metastasis
Source: EMBO Mol Med. 2013 Dec 27;6(2):278–94. doi: 10.1002/emmm.201303373 (PMC3927960; doi:10.1002/emmm.201303373)
Supplement: Supplementary file 12 [file emmm0006-0278-sd12.pdf]

Supplementary Table S2. Clinicopathologic features of the 95 primary breast tumors assayed for ADAM8 protein expression by ELISA (corresponding to Figure 1B).

Tumor characteristic	***N (%)***
**Age**	
≤ 50 yrs	9 (9.5)
> 50 yrs	85 (89.5)
Unknown	1 (1.1)
**Size**	
≤ 2 cm	19 (20.0)
> 2 cm	73 (76.8)
Unknown	3 (3.2)
**Grade**	
1 & 2	38 (40.0)
3	50 (52.6)
Unknown	7 (7.4)
**ER Status**	
Negative	30 (31.6)
Positive	60 (63.2)
Unknown	5 (5.2)
**PR Status**	
Negative	56 (59.0)
Positive	31 (32.6)
Unknown	8 (8.4)
**HER2 Status**	
Negative	16 (16.8)
Positive	2 (2.1)
Unknown	77 (81.1)
**Nodal Status**	
Negative	33 (34.7)
Positive	58 (61.1)
Unknown	4 (4.2)
**Histology**	
Ductal	72 (75.8)
Lobular	15 (15.8)
Ductal&Lobular	6 (6.3)
Unknown	2 (2.1)
