# Supplementary material for: ADAM8 expression in invasive breast cancer promotes tumor dissemination and metastasis
Source: EMBO Mol Med. 2013 Dec 27;6(2):278–94. doi: 10.1002/emmm.201303373 (PMC3927960; doi:10.1002/emmm.201303373)
Supplement: Supplementary file 13 [file emmm0006-0278-sd13.pdf]

Supplementary Table S3. Clinical characteristics of the 30 breast cancer sera analyzed by ADAM8 ELISA in this study (corresponding to Figure 1C).

Tumor characteristic	***N (%)***
**Age**	
≤ 50 yrs	8 (26.7)
> 50 yrs	19 (63.3)
Unknown	3 (10.0)
**Size**	
≤ 2 cm	10 (33.3)
> 2 cm	9 (30.0)
Unknown	11 (36.7)
**Grade**	
1 & 2	13 (43.3)
3	13 (43.3)
Unknown	4 (13.4)
**ER Status**	
Negative	6 (20.0)
Positive	21 (70.0)
Unknown	3 (10.0)
**Nodal Status**	
Negative	13 (43.3)
Positive	7 (23.3)
Unknown	10 (33.3)
**Histology**	
Ductal	21 (70.0)
Lobular	4 (13.3)
Ductal&Lobular	1 (3.3)
Unknown	4 (13.3)
