# Supplementary material for: ADAM8 expression in invasive breast cancer promotes tumor dissemination and metastasis
Source: EMBO Mol Med. 2013 Dec 27;6(2):278–94. doi: 10.1002/emmm.201303373 (PMC3927960; doi:10.1002/emmm.201303373)
Supplement: Supplementary file 14 [file emmm0006-0278-sd14.pdf]

Supplementary Table S4. Clinical characteristics of the primary and metastatic breast tumors assayed for ADAM8 protein expression by immunohistochemistry, including 37 Ductal Carcinoma *In situ* (DCIS), 50 Triple-Negative Breast Cancers (TNBCs) and 56 metastases (corresponding to Figure 1E). N(%) is shown.

Tumor characteristic	***DCIS***	***TNBC***	***Metastasis***
**Age**			
≤ 50 yrs	12 (32.4)	13 (26.0)	0
> 50 yrs	25 (67.6)	37 (74.0)	0
Unknown	0	0	56 (100.0)
**Grade**			
1 & 2	0	14 (28.0)	4 (7.1)
3	0	35 (70.0)	9 (16.1)
Unknown	37 (100.0)	1 (2.0)	43 (76.8)
**ER Status**			
Negative	0	50 (100.0)	6 (10.7)
Positive	0	0	7 (12.5)
Unknown	37 (100.0)	0	43 (76.8)
**HER2 Status**			
Negative	0	50 (100.0)	8 (14.3)
Positive	0	0	5 (8.9)
Unknown	37 (100.0)	0	43 (76.8)
**Nodal Status**			
Negative	0	33 (66.0)	6 (10.7)
Positive	0	16 (32.0)	5 (8.9)
Unknown	37 (100.0)	1 (2.0)	45 (80.4)
**Histology**			
Ductal	37 (100.0)	44 (88.0)	12 (21.4)
Lobular	0	3 (6.0)	1 (1.8)
Others	0	3 (6.0)	0
Unknown	0	0	43 (76.8)
