# Supplementary material for: ADAM8 expression in invasive breast cancer promotes tumor dissemination and metastasis
Source: EMBO Mol Med. 2013 Dec 27;6(2):278–94. doi: 10.1002/emmm.201303373 (PMC3927960; doi:10.1002/emmm.201303373)
Supplement: Supplementary file 15 [file emmm0006-0278-sd15.pdf]

Supplemental Methods

Cell Lines and Culture Conditions

Human TNBC cell lines (MDA-MB-231, Hs578T, MDA-MB-468, BT549), non-tumoral mammary epithelial cell line MCF-10A, embryonic kidney cell line HEK293 and umbilical vein endothelial cell line HUVEC were purchased from the American Type Culture Collection (ATCC) and maintained in medium recommended by ATCC. Inflammatory breast cancer cell line SUM149 was kindly provided by Stephen Ethier (University of Michigan Medical School, Ann Arbor, MI) and maintained as previously described (Forozan et al, 1999). MDA-MB-231-derived LM1 and LM2 cells, kindly provided by Joan Massagué (Memorial Sloan-Kettering Cancer Center, New York, NY), were isolated *in vivo* for their ability to metastasize to the lung (Minn et al, 2005). Stable clones of *ADAM8* shRNA (shA8) and *Control* shRNA (shCtrl) MDA-MB-231 cells, expressing GFP, were established after transfection of either *ADAM8* shRNA or scramble control vector (Origene), respectively, using LTX Lipofectamine (Invitrogen), selection with 1 μg/ml puromycin (Sigma), and limiting dilution. MDA-MB-231 clones were authenticated using short tandem repeat analysis (Genetica DNA Laboratories), which demonstrated 100% identity with the MDA-MB-231 cell line from ATCC.

Western blotting

Whole-cell extracts (WCEs) from cells in culture or frozen homogenized tumor tissue were prepared using RIPA buffer supplemented with protease and phosphatase inhibitors (Pierce), as well as EDTA and phenanthroline to inhibit the autocatalytic activity of ADAM8. Samples (25 μg) were subjected to immunoblotting as previously described (Romagnoli et al, 2012). Antibodies against ADAM8 were purchased from Millipore (AB19017) and LSBio (B4068). Antibody against VEGF-A was purchased from R&D Systems (AF293NA). Antibodies against

β-Actin (AC-15) and β-Tubulin (TUB 2.1) were purchased from Sigma. For Western blot analysis of released VEGF-A, serum-free medium incubated with cells for 16 h was collected, centrifuged at 4,000 rpm for 10 min to remove cell debris and concentrated using centrifugal 10K filters (Amicon Ultra-15). Volumes of the conditioned medium corresponding to 2.5×10^5 cells were subjected to immunoblotting using a VEGF-A antibody (R&D Systems, MAB293).

ATP assay

As a measure of cellular metabolism and therefore cell growth, ATP levels were assessed in 3×10^3 MDA-MB-231 cells using a luminescence ATP detection assay (Perkin Elmer), as described previously (Mineva et al, 2009). Average ATP levels are presented relative to control condition set to 1 (Mean ± S.D. from 3 independent experiments).

Soft agar and Matrigel outgrowth assays

For soft agar assays, 5×10^4 cells in a mix of 0.4% Bacto Agar (BD Biosciences) in complete media were plated in triplicate on 6-well dishes pre-coated with a 1:1 mix of 2x DMEM medium supplemented with 10% FBS and 1.6% Bacto Agar. Cells were fed 3 times per week with complete medium. After 8-12 days, cells were stained overnight with 0.2 mg/ml iodonitrotetrazolium chloride (Sigma) and photographed at 10x magnification. Colonies with diameters of >20 μm were counted using ImageJ software (NIH). Matrigel outgrowth assays were carried out as described previously (Belguise et al, 2007), using 5×10^3 cells plated in duplicate on 24-well plates. Cultures were incubated for 4-7 days and photographed at 20x magnification.

Migration/invasion and transendothelial migration assays

Migration and invasion assays were performed in triplicate using polycarbonate filter Transwells (Costar) with 8-μm-diameter pores, without precoating or precoated with growth factor-reduced

Matrigel (BD Biosciences, 356231), respectively. Transendothelial migration assays were performed as above with Transwells precoated with HUVECs that had been allowed to grow until a confluent monolayer was achieved. Suspensions of 1 x 10^5 tumor cells were layered in the upper compartments of the Transwell and incubated at 37°C. After 24 h, cells that migrated or invaded to the lower side of the filter were evaluated by crystal violet staining and OD_{570nm} determination. The control condition (siCtrl, shCtrl, or isotype antibody control) was set to 100% and the mean ± S.D. from 3 independent experiments is given.

Tube formation assay

Tube formation assays were performed as described (Kim et al, 2007). Briefly, 2 x 10^4 HUVEC cells were plated, in triplicate, on glass chambers coated with growth factor reduced Matrigel. Cells were incubated in 200 µl EBM-2 Basal Medium supplemented with 1% FBS and concentrated conditioned medium from MDA-MB-231 clones (volume corresponding to 5 x 10^5 tumor cells) or conditioned media in the absence of tumor cells. Following incubation for 8 h, HUVECs were stained with Calcein AM solution (1 mg/ml) according to the manufacturer's instructions (PromoCell, PK-CA707-80011-2) for 15-30 min at 37°C, and 3 random fields/well were photographed (40x magnification). Incomplete networks were excluded and numbers of closed networks (polygons) of vessel-like tubes and branch points counted in 3 fields (n = 9). The average percentage relative to control samples is presented ± S.D. from a representative of 3 independent experiments.

ADAM8 metalloproteinase activity assay

HEK-293 cells were plated at 5 x 10^5 cells/ml in P60 dishes. After 24 h, cultures were co-transfected with 1 µg of plasmid DNA encoding C-terminal HA-tagged CD23 (CD23 membrane isoform b, purchased from Addgene, deposited by Zena Werb, University of California, CA) and

1 μg vector DNA expressing either ADAM8 full-length or remnant form, or empty vector (EV) pCDNA3.1 Version B DNA. The medium was replaced after 6 h with culture medium without FBS. After 16 h, the serum-free media was harvested and centrifuged to remove cell debris, while cells were trypsinized and counted. The supernatants were concentrated using Amicon-ultra 4 centrifugal filter units (Millipore) and volumes corresponding to equal cell numbers were assessed for cleaved CD23 using Western blotting. WCEs from lysed cells were further assessed for ADAM8 levels.

Detection of CTCs.

Diluted whole blood samples were flowed in microfluidic devices, each of which had five single 50 μm (width) by 70 μm (height) and 10 mm (length) channels. To flow cells in a microfluidic channel for light scattering and fluorescence emission measurements, the outlet tubing was placed into Eppendorf tubes and the heparinized blood suspension was drawn by a syringe pump (Harvard Apparatus) at a flowrate of 6 μl min^{-1}. Operating at a constant pressure, a 6 μl min^{-1} set flowrate calibrated for water yielded an actual value of 4.5 μl min^{-1} due to the increased viscosity of blood. Channels were pre-wetted by flowing PBS and the blood sample flow was allowed to stabilize prior to data acquisition. Of the initial ~100 μl of whole blood collected, 34±17, 55±17, and 57±9 μl were analyzed for non-tumor bearing, *shControl*, and *shADAM8* mice, respectively. For the final 33 day timepoint, 100 μl of blood was analyzed.

Counts positive for GFP fluorescence were detected in the 500 to 590 nm "green" channel following excitation with 488 nm light and were time-correlated with 405 and 488 nm scattering values of sufficient intensity. Within the volume analyzed, leukocytes and CTCs constituted ~0.5% of the acquired backscattered and fluorescent light data stream. Using a 25 kHz sampling frequency, the lower 99.5% of the intensity values defined the mean and standard

deviation of the background signal. Individual peaks were demarcated by 6 to 7 data points and the maximum value defined the intensity used to determine whether the count contained sufficiently intense scattering and fluorescence values for CTC identification. Within the previously developed Matlab program, the intensity threshold was defined based on the number of standard deviations above the mean of the background signal. The number of standard deviations was 4, 4, and 5 for the 405, 488, and 500-590 nm channels, respectively. Of the non-tumor bearing samples assayed, a concentration of mean ± S.D. μl^{-1} green autofluorescent cells were detected and subtracted from the detected counts in tumor-bearing samples, yielding the reported CTC concentration values.

References

Belguise K, Guo S, Sonenshein GE (2007) Activation of FOXO3a by the green tea polyphenol epigallocatechin-3-gallate induces estrogen receptor alpha expression reversing invasive phenotype of breast cancer cells. Cancer Res 67:5763-5770

Forozan F, Veldman R, Ammerman CA, Parsa NZ, Kallioniemi A, Kallioniemi OP, Ethier SP (1999) Molecular cytogenetic analysis of 11 new breast cancer cell lines. Br J Cancer 81:1328-1334

Kim HJ, Chung H, Yoo YG, Kim H, Lee JY, Lee MO, Kong G (2007) Inhibitor of DNA binding 1 activates vascular endothelial growth factor through enhancing the stability and activity of hypoxia-inducible factor-1alpha. Mol Cancer Res 5:321-329

Mineva ND, Wang X, Yang S, Ying H, Xiao ZX, Holick MF, Sonenshein GE (2009) Inhibition of RelB by 1,25-dihydroxyvitamin D3 promotes sensitivity of breast cancer cells to radiation. J Cell Physiol 220:593-599

Minn AJ, Gupta GP, Siegel PM, Bos PD, Shu W, Giri DD, Viale A, Olshen AB, Gerald WL, Massague J (2005) Genes that mediate breast cancer metastasis to lung. Nature 436:518-524

Romagnoli M, Belguise K, Yu Z, Wang X, Landesman-Bollag E, Seldin DC, Chalbos D, Barille-Nion S, Jezequel P, Seldin ML*, et al* (2012) Epithelial-to-mesenchymal transition induced by TGF-beta1 is mediated by Blimp-1-dependent repression of BMP-5. Cancer Res 72:6268-6278

van de Vijver MJ, He YD, van't Veer LJ, Dai H, Hart AA, Voskuil DW, Schreiber GJ, Peterse JL, Roberts C, Marton MJ*, et al* (2002) A gene-expression signature as a predictor of survival in breast cancer. N Engl J Med 347:1999-2009
