# Supplementary material for: ADAM8 expression in invasive breast cancer promotes tumor dissemination and metastasis
Source: EMBO Mol Med. 2013 Dec 27;6(2):278–94. doi: 10.1002/emmm.201303373 (PMC3927960; doi:10.1002/emmm.201303373)
Supplement: Supplementary file 16 [file emmm0006-0278-sd16.pdf]

SUPPLEMENTARY INFORMATION

"ADAM8 expression in invasive breast cancer promotes tumor dissemination and metastasis" *by Romagnoli et al.*

Supplementary Figures:

Supplementary Fig S1. *ADAM8* mRNA levels correlate with clinicopathological variables of human breast tumors.

Supplementary Fig S2. ADAM8 proform is induced in Hs578T cells grown in 3D-cultures, which co-migrates with the one seen in MDA-MB-231 cells.

Supplementary Fig S3. Ectopic ADAM8 expression rescues the invasive phenotype of stable ADAM8 knockdown cells.

Supplementary Fig S4. ADAM8 knockdown tumors fail to grow beyond a palpable size and are poorly vascularized in a mammary fat pad mouse model.

Supplementary Fig S5. HIF-1α is induced in breast cancer cell lines under hypoxia.

Supplementary Fig S6. Ectopic expression of full-length ADAM8 and remnant form in stable ADAM8 knockdown cells.

Supplementary Fig S7. siRNA-mediated knockdown of ADAM8 in MDA-MB-231 cells reduces their ability to adhere to and transmigrate through HUVECs.

Supplementary Fig S8. ADAM8 is not detected in HUVECs.

Supplementary Fig S9. Anti-ADAM8 antibody inhibits the metalloprotease activity of ADAM8.

Supplementary Fig S10. *VEGF-A* mRNA levels are unchanged in stable ADAM8 knockdown cells.

Supplementary Tables:

Supplementary Table S1. Multivariate analysis of *ADAM8* mRNA as a predictor in breast cancer.

Supplementary Table S2. Clinical characteristics of the human primary breast tumors analyzed by ADAM8 ELISA.

Supplementary Table S3. Clinical characteristics of the sera from breast cancer patients analyzed by ADAM8 ELISA.

Supplementary Table S4. Clinical characteristics of the primary and metastatic samples from breast cancer patients analyzed for ADAM8 by immunohistochemistry.

Supplementary Methods:

Cell Lines and Culture Conditions

Western blotting

ATP assay

Soft agar and Matrigel outgrowth assays

Migration/invasion and transendothelial migration assays

Tube formation assay

ADAM8 metalloproteinase activity assay

Detection of CTCs

References
