# Supplementary material for: ADAM8 expression in invasive breast cancer promotes tumor dissemination and metastasis
Source: EMBO Mol Med. 2013 Dec 27;6(2):278–94. doi: 10.1002/emmm.201303373 (PMC3927960; doi:10.1002/emmm.201303373)
Supplement: Supplementary file 17 [file emmm0006-0278-sd17.pdf]

ADAM8 expression in invasive breast cancer promotes tumor dissemination and metastasis

Mathilde Romagnoli, Nora D. Mineva, Michael Polmear, Catharina Conrad, Srimathi Srinivasan, Delphine Loussouarn, Sophie Barillé-Nion, Irene Georgakoudi, Áine Dagg, Enda W. McDermott, Michael J. Duffy, Patricia M. McGowan, Uwe Schlomann, Maddy Parsons, Jörg W. Bartsch, and Gail E. Sonenshein

Corresponding author: Gail Sonenshein, Tufts University School of Medicine

Review timeline:

Submission date:	04 June 2013
Editorial Decision:	06 June 2013
Resubmission:	08 August 2013
Editorial Decision:	28 August 2013
Revision received:	17 October 2013
Editorial Decision:	04 November 2013
Revision received	13 November 2013
Accepted:	15 November 2013

Transaction Report:

(Note: With the exception of the correction of typographical or spelling errors that could be a source of ambiguity, letters and reports are not edited. The original formatting of letters and referee reports may not be reflected in this compilation.)

Editor: Roberto Buccione

1st Editorial Decision 06 June 2013

Thank you for the submission of your manuscript " ADAM8 expression in invasive breast cancer promotes tumor dissemination and metastasis ".

I have now had the opportunity to carefully read your paper and the related literature and I have also discussed it with my colleagues. I am afraid that we concluded that the manuscript is not well suited for publication in EMBO Molecular Medicine and have therefore decided not to proceed with peer review.

You find that ADAM8 is abundantly expressed in breast cancer and especially in triple-negative BC (TNBC) compared to normal tissue. You also show that ADAM8 promoted an aggressive phenotype in TNBC cells in vitro and in a mouse mammary fat pad model, and that TNBC cells with ADAM8 knockdown grew and metastasized poorly. We appreciate that ADAM8 appears to stimulate angiogenesis through the release of pro-angiogenic factors including VEGF-A and regulates subsequentextra/intravasation steps.

Your work is in principle within the scope of EMBO Molecular Medicine but unfortunately we feel that, as it is, the manuscript falls short on an important aspect: the current lack of validation of ADAM8 as a potential target in a relevant model (such as for instance with antibodies and/or inhibitors), which prevents us from considering this manuscript further.

However, if you can provide this additional information in a future version, I would commit to sending it out for peer review.

I am sorry that I could not bring better news this time.

Resubmission 08 August 2013

Enclosed please find an updated version of a manuscript entitled "**ADAM8 expression in invasive breast cancer promotes tumor dissemination and metastasis**" that we are submitting as an article to EMBO Molecular Medicine. In this new version, we have included the requested data showing that treatment with an ADAM8 antibody reduces tumor burden and metastases in an orthotopic mouse model, which provides a proof of concept validating ADAM8 protein as a therapeutic target for triple-negative breast cancer.

Triple-negative breast cancer (TNBC) is highly aggressive and lacks targeted therapies due to the absence of hormone and HER2 receptors. TNBC frequently recurs, responds only poorly to subsequent chemotherapy and thus accounts for approximately 25% of the half million yearly deaths worldwide from breast cancer. In this study, for the first time, the metalloproteinase-disintegrin protein ADAM8 is identified and validated as a novel target for treatment of TNBC.

A few years ago, we discovered a RelB NF-kB/Blimp-1 pathway that was aberrantly expressed in breast cancers and promoted their invasive phenotype. Unfortunately, both RelB and Blimp-1 play important functions in the immune system and thus did not represent safe therapeutic targets. Therefore, we have been walking downstream of this pathway to identify a druggable target for the treatment of aggressive breast tumors and identified ADAM8. ADAM8 is a cell surface transmembrane protein that mediates cell adhesion and shedding of ligands, receptors and extracellular matrix components. ADAM8-deficent mice do not show any developmental or pathological defects, indicating that ADAM8 is non-essential under physiological conditions. In the present manuscript, our team collaborated closely to characterize the expression of ADAM8 in breast cancer [D Loussouarn and S Barillé-Nion (Nantes); MJ Duffy and P McGowan (Dublin)] and to elucidate the roles of ADAM8 in aggressive breast cancers [J Bartsch (Marburg); I Georgakoudi (Boston); M Parsons (London)].

Here, we demonstrate for the first time that ADAM8 is abundantly expressed in primary and metastatic breast tumors, especially in TNBC, and plays essential functions in tumor growth and dissemination. In particular, ADAM8 is required for angiogenesis (via the release of several pro-angiogenic growth factors including VEGF-A) and tumor cell spreading (via activation of b1-integrin which is necessary for tumor cell intra/extravasation). Importantly, an ADAM8 antibody-based therapeutic approach is shown here to effectively reduce tumor growth (burden), dissemination (metastases), and angiogenesis in an orthotopic mouse model.

Our major findings are summarized below:

a. Using ELISA, immunohistochemistry and microarray databases, ADAM8 was found to be abundantly expressed in aggressive breast cancers and their metastases, specifically in TNBC (ER-, PR-, HER2-), and associated with poor clinical outcomes.

b. Knockdown of ADAM8 in TNBC cells *in vitro* reduced their invasive phenotype but not their growth in 2D cultures.

c. In an orthotopic mouse mammary model, stable ADAM8 knockdown in TNBC cells led to a profound reduction in growth, the numbers of Circulating Tumor Cells and the incidence of brain metastases, consistent with the patient data.

d. ADAM8 expression is induced by hypoxia in mice and in patient tumors.

e. ADAM8 knockdown in TNBC cells profoundly reduced their ability to promote tube formation by human endothelial cells and to release several pro-angiogenic factors, including VEGF-A. Consistently, the vascularization surrounding shADAM8 tumors was reduced as measured by CD31 staining.

f. ADAM8 was required for b1-integrin activation, and for TNBC cell binding to and migration across an endothelial cell layer *in vitro*. Importantly, this inhibitory effect was recapitulated by addition of a monoclonal antibody targeting the extracellular domain (ectodomain) of ADAM8.

g. In an orthotopic mouse mammary model of TNBC, tumor burden, metastasis and angiogenesis were all significantly reduced upon treatment with 0.5 mg/kg ADAM8 ectodomain antibody.

In summary, despite advances in breast cancer treatment, TNBC frequently progresses to invasive carcinoma with fatal metastatic spread particularly in younger women. Overall, our studies present compelling evidence that ADAM8 represents a promising novel target for treatment of patients with TNBC and suggest that an antibody-based therapy would be successful.

Our collaborator Joerg W. Bartsch has conducted a complementary study on pancreatic cancer, which was previously shown to express ADAM8. His team has demonstrated that targeting ADAM8 with a specific cyclic peptide inhibitor potently reduces tumor burden and development of metastasis in two mouse models of aggressive pancreatic tumorigenesis, which further confirms the feasibility of targeting ADAM8 *in vivo*. This manuscript, which is not overlapping, is under review at the Journal of Experimental Medicine.

These studies are not under consideration at any other journal. The authors have no competing interests. As potential editor, we suggest either Alan Ashworth or Kari Alitalo. As potential reviewers, we suggest Professors Dylan Edwards (UEA, Norwich, UK), Hans-Peter Altevogt (German Cancer Research Center, Cell Adhesion and Metastasis unit) and Gary Stein (Chair of the Department of Biochemistry, University of Vermont) for their expertise in cancer, extracellular matrix, and metalloproteinases.

Your consideration of our manuscripts is greatly appreciated.

2nd Editorial Decision 28 August 2013

Thank you for the submission of your manuscript to EMBO Molecular Medicine. We have now received reports from the three Reviewers whom we asked to evaluate your manuscript

You will see that all three Reviewers are generally supportive of your work and underline its considerable potential interest. They do however express a few nicely complementary concerns that require your attention and action. For this reason, publication of the paper cannot be considered at this stage.

Reviewer 1 notes the limited rescue in the ADAM8 knock-down experiments and suggests that a second independent shRNA would be required to exclude potential off-target effects. S/he also questions your choice of selecting an MDA knock-down clone rather than proceeding with the cell pool.

Reviewer 2 is concerned about the efficacy of the anti-ADAM8 antibody treatment and would like more information on its use and biological effects.

Reviewer 3 is especially concerned about the clinical survival data and suggests stratifying for

triple-negative BC as well as for all BC.

The three Reviewers also mention other important issues that should not prove difficult to address

We would be thus pleased to consider a suitably revised version.

Please note that it is EMBO Molecular Medicine policy to allow a single round of revision only and that, therefore, acceptance or rejection of the manuscript will depend on the completeness of your responses included in the next, final version of the manuscript.

I look forward to receiving your revised manuscript as soon as possible.

***** Reviewer's comments *****

Referee #1 (Remarks):

The authors investigated the metalloprotease-desintegrin ADAM8 in breast cancer. They convincingly reveal that RNA and protein are overexpressed in breast cancer, especially in TNBC, and that high levels correlate with a bad prognosis. They show that ADAM8 knockdown causes migration, invasion and colony forming defects. Moreover, in vivo in a xenograft model they demonstrate that knockdown reduces tumor size and metastasis. Mechanistically, they show that ADAM8 in necessary for tumor blood vessel formation by secreting pro-angiogenic factors, like VEGF. Furthermore, they reveal that ADAM8 facilitates the binding between a breast cancer cell with endothelial cells, a crucial step in the process of metastasis, by activation of b1-integrin on these cells. Finally, using an antibody against b1-integrin they see a significant reduction in primary tumor size and metastasis in the MDA-MB231 xenograft model.

In general it an interesting, solid and good presented piece of work, but there are some points that need to be addressed:

Major point:

Fig4. The authors use 1 shRNA for their experiments, and show only a partial rescue upon overexpression of ADAM8, which is strange considering the high level of activated ADAM8. A second independent shRNA would be more convincing to exclude off target effects. Moreover, it is not clear why they generated cell clones of one particular MDA-MB231 knockdown line (shA8) since previous studies by the Massague lab have shown that individual MDA-231 clones behave differently in metastatic potential. Indeed, the LM1 and LM2 derivatives used in the paper originated from single cell cloning followed by in vivo selection for metastasis to specific organs. Why did they not choose to work with the pool of shA8 KD cells and rescue these with the add-back vector (Fig S3)?

Other points:

Fig1. Could the authors speculate on why ADAM8 is upregulated in breast cancer? Is it mutated or hyperactive?

Fig3. How does ADAM8 knockdown function - do the MB231 and Hs578T cells accumulate in G1 or another phase of the cell cycle? Do they undergo cell death? Do they have any information on this that could be added?

Fig5. Is there anything known on how ADAM8 regulated the expression of the upregulated angiogenic factors?

Fig6F The way figure 6F is presented is difficult to understand. It is not clear from the legend or from the figure which lanes got the b1 antagonist antibody or the HUVECs or shCtrl cells.

Fig7. From the data presented in Fig 5H, one would predict that VEGFA or another of the angiogenic mediators picked up in the screen, would also be decreased in the tumors from the mice treated with the beta1 blocking antibody. Is this the case? Do they have any information on this?

Referee #2 (Comments on Novelty/Model System):

This is a well executed study of potentially high novelty once further understanding of mechanism are elucidated. All the models are appropriate for a pre-clinical study.The medical impact cannot currently be assessed.

This study of ADAM8 shows significant expression in triple negative breast cancers and a correlation with poor outcome. The requirement of ADAM8 for growth, vascularisation and spread of tumours are demonstrated in an orthotopic model. The data appear sound and significant. Some preliminary evidence of potential mechanisms of ADAM8 action is presented which support previous studies.

Specific comments

Fig. 5A legend appears to be incorrect. For ease of reader comprehension Fig.5B legend should also indicate that shRNAs are being used to manipulate ADAM8 levels (ditto other Fig. legends). There are no details of the shRNAs to ADAM8 in the manuscript.
p.10 ADAM8 and the 'release' of pro-angiogenic factors. Is there any evidence that this process involved the proteolytic function of ADAM8 (as cited in the Introduction)? This is not discussed here or in the Discussion but is a relevant issue and needs to be addressed.
Fig.6A needs a better explanatory legend.
Fig.7A, activity of R&D MAB1301 against ADAM8 seems to be rather poor compared to the previous ADAM8 shRNA KD data. However, the amount of antibody administered was rather low. Was a dose study carried out? Did higher levels of MAB1301 show more efficient abrogation of tumour development? Is MAB1301 known to inhibit ADAM8 proteolytic activity?

Referee #2 (Remarks):

The study complements one carried out in a GEMM model of pancreatic cancer by the bartsch and Tuveson groups that is submitted for publication but apparently not yet accepted.

Referee #3 (Comments on Novelty/Model System):

No concerns about models used.

My primary concern is in the interpretation of the clinical survival data.

Referee #3 (Remarks):

ADAM8 is a transmembrane protein that belongs to the ADAM (a disintegrin and metalloprotease) family. Here, the investigators report that ADAM8 protein is expressed in 34% triple-negative breast

cancers (TNBCs) (versus not detectable in normal breast tissue) and its level correlates with disease free and overall survival. The investigators provide evidence that ADAM8 promotes angiogenesis and metastasis in mouse xenografts.

This is a compelling and well-crafted manuscript that investigates the mechanism of ADAM8 signaling in breast carcinogenesis. The manuscript is well organized and experiments well designed and described. The investigators provide evidence that ADAM8 is an important regulator of poor prognosis breast cancer.

Suggestions for strengthening the manuscript:

1. It would be useful to know the frequency of ADAM8 expression in breast cancer subtypes other than TNBC.

2. The investigators postulate a role for ADAM8 in TNBC. Survival curves in Figure 1.F and G should be stratified for TNBC as well as for all breast cancers. This analysis would better differentiate whether ADAM8 predicts poor prognosis (as opposed to ADAM8 being expressed in TNBC more frequently and TNBC carrying a poor prognosis relative to other breast cancer subtypes).

3. The reason(s) for differences in the migration of the pro-form of ADAM8 in Hs578T is speculative and should be presented as such.

4. The statement that "... ADAM8 contributed to the maintenance of a mesenchymal phenotype of TNBC cells in 3D-culture.", in the 1st paragraph of the discussion, is poorly supported by the data presented.

5. The Discussion is well written but would be improved by additional focusing (25-35% reduction).

1st Revision - authors' response 17 October 2013

October 17, 2013

Roberto Buccione, PhD
Editor, EMBO Molecular Medicine
EMBO Molecular Medicine
Re: EMM-2013-03373

Dear Dr. Buccione:

Enclosed please find a revised version of our manuscript entitled "**ADAM8 expression in invasive breast cancer promotes tumor dissemination and metastasis**" that we are submitting as an article to EMBO Molecular Medicine. We were very pleased to learn that the reviewers found our manuscript was "compelling and well-crafted", "solid" and that the experiments were "well designed and described". We thank the referees for their positive comments and for their constructive suggestions. We have responded to the three major reviewer issues discussed in your summary and addressed all of the individual comments of the referees below. In particular, in this revised manuscript we have (1) added a new orthotopic mouse resection experiment using a higher dose of the ADAM8 ectodomain antibody that demonstrates the effectiveness of targeting ADAM8 to reduce tumor dissemination and further addresses concerns about potential off-target effects and antibody dose; (2) demonstrated the role of the metalloproteinase domain in release of VEGF-A; (3) demonstrated that the anti-ADAM8 antibody reduces VEGF-A levels in tumors and metalloproteinase activity *in vitro*; (4) confirmed that high *ADAM8* levels correlate with poor breast cancer patient outcome. Overall our revised manuscript provides compelling validation of ADAM8 as a novel target for treatment of triple-negative breast cancer (TNBC).

Major comments
Reviewer 1 notes the limited rescue in the ADAM8 knock-down experiments and suggests that a second independent shRNA would be required to exclude potential off-target effects. S/he also questions your choice of selecting an MDA knock-down clone rather than proceeding with the cell pool.

Our decision to use clones was based on the observation that ADAM8 has the ability to function through paracrine fashion, e.g., via release/shedding of pro-angiogenic factors and remodeling of the ECM. Thus, we felt it was essential to achieve a very effective knockdown for testing the role of ADAM8 in an animal experiment. We verified that the shADAM8 cells displayed similar *in vitro* phenotypic changes as seen with two independent ADAM8 siRNAs, and have performed additional experiments that confirm this point.

The rescue experiment, which was limited by the transfection efficiency of these cells (40%), demonstrated the ability of ectopic ADAM8 to appropriately restore the invasive phenotype (as shown in Fig S3). Notably, the importance of ADAM8 *in vivo* was confirmed using an antibody against the ADAM8 extracellular domain at the time of cell implantation. The ADAM8 ectodomain antibody caused a significant reduction in tumor growth and dissemination compared to an isotype control IgG. In a new orthotopic mouse experiment

136 Harrison Avenue, Boston, MA 02111-1800 | TEL: 617.636.4091 | FAX: 617.636.2409 | Gail.Sonenshein@tufts.edu

added to this revised version, we have extended these findings to look at the effects of the antibody on metastases from established tumors using a neoadjuvant resection protocol and a higher antibody dose (1.5 mg/kg, please see comments to Reviewer 2). Once palpable tumors were detected (~2 weeks after cell implantation), mice were treated twice with either an anti-ADAM8 antibody or an isotype control for one week and then tumors were resected. After a 5-week treatment regimen, a profound reduction in metastases to the brain and lungs was observed with anti-ADAM8 antibody treatment. These studies confirm that targeting ADAM8 with an antibody-based approach reduces dissemination of TNBC tumors.

Reviewer 2 is concerned about the efficacy of the anti-ADAM8 antibody treatment and would like more information on its use and biological effects.

We had originally planned on using the same dose that was successful in one of the initial tests of Herceptin in preventing tumor growth (1 mg/kg) (Baselga et al, Cancer Res 1998). Unfortunately, the actual dose was half of that (0.5 mg/kg) due to a production error by R&D Systems, which we only learned about after the experiment was completed. Importantly, the findings obtained with 0.5 mg/kg ADAM8 antibody were significant and similar to the data obtained with 0.3 mg/kg Herceptin antibody. As we were using a commercial antibody, we decided in favor of testing the effects of the antibody in a more translationally relevant resection experiment where we could look at its ability to affect metastasis of pre-existing tumors. In this resection experiment, we tried to mimic the newer neoadjuvant protocols of initiating treatment prior to surgery. Also, we raised the antibody dose to 1.5 mg/kg. Thus, tumors were grown to approximately 60-80 mm^3, a size at which angiogenesis has typically initiated. Mice were then injected with antibody twice in a span of one week prior to tumor resection. An additional dose was given the day of tumor resection and then treatment was continued twice weekly for an additional 5 weeks. A profound decrease in occurence and numbers of metastases to both the brain and lungs was seen with the anti-ADAM8 antibody treatment (new panels Fig 7F and 7G).

Reviewer 3 is especially concerned about the clinical survival data and suggests stratifying for triple-negative BC as well as for all BC.

We considered doing this analysis but as TNBC constitutes only approximately 15% of all breast cancers, the van de Vijver dataset included only a small number of TNBC samples (i.e., 41). It would not be appropriate to use such a small number of patients to identify a possible prognostic effect. In response to the reviewer, however, we re-ran the analysis after removing the TNBC samples from the total population and high *ADAM8* expression was still significantly associated with a poorer prognosis in the remaining breast cancer patients (overall survival using 75th percentile cutoff in the dataset minus basal samples: P = 0.003, HR = 2.41 CI = 1.36-4.27).

Referee #1 *(Remarks):*

The authors investigated the metalloprotease-disintegrin ADAM8 in breast cancer. They convincingly reveal that RNA and protein are overexpressed in breast cancer, especially in TNBC, and that high levels correlate with a bad prognosis. They show that ADAM8 knockdown causes migration, invasion and colony forming defects. Moreover, in vivo in a xenograft model they demonstrate that knockdown reduces tumor size and metastasis. Mechanistically, they show that ADAM8 in necessary for tumor blood vessel formation by secreting pro-angiogenic factors, like VEGF. Furthermore, they reveal that ADAM8 facilitates the binding between a breast cancer cell with endothelial cells, a crucial step in the process of metastasis, by activation of β1-integrin on these cells. Finally, using an antibody against b1-integrin they see a significant reduction in primary tumor size and metastasis in the MDA-MB231 xenograft model.

In general it an interesting, solid and good presented piece of work, but there are some points that need to be addressed:

Major point:
Fig4. The authors use 1 shRNA for their experiments, and show only a partial rescue upon overexpression of ADAM8, which is strange considering the high level of activated ADAM8. A second independent shRNA would be more convincing to exclude off target effects. Moreover, it is not clear why they generated cell clones of one particular MDA-MB231 knockdown line (shA8) since previous studies by the Massague lab have shown that individual MDA-231 clones behave differently in metastatic potential. Indeed, the LM1 and LM2 derivatives used in the paper originated from single cell cloning followed by in vivo selection for metastasis to specific organs. Why did they not choose to work with the pool of shA8 KD cells and rescue these with the add-back vector (Fig S3)?

There might be a slight misunderstanding on how the second mouse experiment was performed. We actually used an anti-ADAM8 ectodomain antibody with MDA-MD-231 shCtrl cells and not the β1-integrin antibody in this analysis. Importantly, the ADAM8 antibody caused a significant reduction in tumor growth, tumor burden and metastasis - in comparison to an isotype matched control IgG2B - similar to the ADAM8 shRNA although to a somewhat lesser extent. In a new neoadjuvant resection experiment using the same ADAM8 antibody, we have observed a profound reduction in metastases to both the brain and the lungs (Fig 7F-G). In addition with respect to off-target effects, the ADAM8 shRNA covers the same target sequence as the ADAM8 siRNA #1 used in this study. We have obtained similar data *in vitro* using this shRNA and two specific ADAM8 siRNAs (#1 and #2) in terms of inhibition of invasive phenotype in 3D-culture, without changes in cell growth on plastic (2D), which suggest that our observations are specific to ADAM8 knockdown. We have now extended these studies using the two ADAM8 siRNAs and observed similar decreases in cancer cell adhesion to HuVECs and transendothelial cell migration (new Supplementary Fig S7). Finally, the partial rescue observed after ectopic expression of ADAM8 can be explained by the 40% plasmid transfection efficiency of MDA-MB-231 cells. With this moderate extent of transfection efficiency, it would be impossible to obtain a higher rescue of the migratory and invasive abilities of these cells. Together, our data quite strongly indicate that the observed *in vitro* and *in vivo* effects of the ADAM8 shRNA are indeed due to knockdown of ADAM8.

Our decision to use clones was based on the observation that ADAM8 has the ability to function through trans interactions and in a paracrine fashion, e.g., via release/shedding of pro-angiogenic factors and remodeling of ECM. Thus, we felt it was essential to achieve a very effective knockdown for testing the role of ADAM8 in an animal experiment. Mixed populations were initially isolated using 4 different ADAM8 shRNAs (Origene, Cat. No. TF314948). When screened, the cell pools all showed a reduction in ADAM8 protein amounts, but the extents were moderate suggesting that the cells were heterogeneous in their ADAM8 levels. As the remaining ADAM8 expression in some cells of the population would have the ability to influence the microenvironment and effectively override the effects of ADAM8 knockdown in the other cells, individual clones were isolated and then screened. We selected two shADAM8 clones with effective ADAM8 knockdown and two clones expressing a Control shRNA. The fact that we can rescue the invasive phenotype of these cells with ectopic ADAM8 expression -to the extent expected based on the transfection efficiency of these MDA-MB-231 cells- suggests that the observed effects are indeed due to ADAM8 knockdown. Furthermore, as mentioned above, similar effects were seen in two orthotopic models with the anti-ADAM8 antibody *in vivo* and with two siRNAs *in vitro* indicating that the effects we are seeing are not due to off-target events.

Other points:
Fig1. Could the authors speculate on why ADAM8 is upregulated in breast cancer? Is it mutated

or hyperactive?

We could find no evidence of *ADAM8* gene amplification in breast cancer on either the Oncomine or the Tumorscape website from the Broad Institute. Interestingly, several pathways commonly dysregulated in aggressive malignancies have been identified that induce *ADAM8* mRNA levels, e.g., EGFR (Sriraman et al, Biol Reprod 2008), K-Ras (JWB, personal data) and RelB NF-κB (GES, personal data), suggesting that ADAM8 is part of the transformation cascade. Mutation of ADAM8 has also been implicated by Lehtimäki and coworkers. This group has shown that carriers of particular SNPs in the *ADAM8* allele have higher expression levels of ADAM8 and an increased risk of advanced atherosclerosis and fatal myocardial infarction (Levula et al, Ann Med 2009, Raitoharju et al, Atherosclerosis 2011). Due to space limitations, we have only added a brief discussion of these possibilities (p. 17).

Fig3. How does ADAM8 knockdown function - do the MB231 and Hs578T cells accumulate in G1 or another phase of the cell cycle? Do they undergo cell death? Do they have any information on this that could be added?

In 2D- and 3D-culture, no differences in cell death (trypan blue) or proliferation (ATP and FACS/cell cycle analyses) were observed upon ADAM8 knockdown using siRNAs or shRNA. Consistently, in a 3D-Matrigel outgrowth assay, we observed that breast cancer cells with ADAM8 knockdown were able to form colonies which can still grow quite large but lack invasive branches. This argues against an effect of ADAM8 knockdown on cell survival or cell cycle.

Fig5. Is there anything known on how ADAM8 regulated the expression of the upregulated angiogenic factors?

This is an interesting question. However, nothing was available in the literature on this topic. Notably, knockdown of ADAM8 in MDA-MB-231 cells did not affect *VEGF-A* mRNA levels but decreased VEGF-A protein levels in the conditioned medium. One major form of VEGF-A regulation occurs through proteolytic cleavage from cell surface or ECM depots. Thus, in response to your question, we tested the role of the full-length ADAM8 proform vs the remnant form (lacking the metalloprotease domain) in VEGF-A release. Our data indicate that ADAM8 proteolytic activity is required: VEGF-A is released in conditioned medium from the shADAM8 clone after ectopic expression of a vector expressing full-length ADAM8 but not the remnant form. We have included these data in a new panel Fig 5I. Consistently, we also now report that the antibody against ADAM8 reduces tumor associated VEGF-A (see discussion below of Fig 7 (Fig 7E) and ADAM8 protease activity (Supplementary Fig S9).

Fig6F The way figure 6F is presented is difficult to understand. It is not clear from the legend or from the figure which lanes got the β1 antagonist antibody or the HUVECs or shCtrl cells.

We apologize for the difficulty and have modified the figure, legend and text to clarify the experiment.

Fig7. From the data presented in Fig 5H, one would predict that VEGFA or another of the angiogenic mediators picked up in the screen, would also be decreased in the tumors from the mice treated with the beta1 blocking antibody. Is this the case? Do they have any information on this?

This is another very interesting question, which we have now addressed. Protein extracts were prepared from tumors of mice treated with anti-ADAM8 or control antibody, and subjected to Western blot analysis. Tumor associated VEGF-A was significantly reduced in the ADAM8 antibody-treated samples, consistent with the results of the *in vitro* and *in vivo* experiments using ADAM8 knockdown cells. These data are provided in a new panel Fig 7E.

Referee #2 *(Comments on Novelty/Model System):*

This is a well executed study of potentially high novelty once further understanding of mechanism are elucidated. All the models are appropriate for a pre-clinical study. The medical impact cannot currently be assessed.

This study of ADAM8 shows significant expression in triple negative breast cancers and a correlation with poor outcome. The requirement of ADAM8 for growth, vascularisation and spread of tumours are demonstrated in an orthotopic model. The data appear sound and significant. Some preliminary evidence of potential mechanisms of ADAM8 action is presented which support previous studies.

Specific comments

Fig. 5A legend appears to be incorrect. For ease of reader comprehension Fig.5B legend should also indicate that shRNAs are being used to manipulate ADAM8 levels (ditto other Fig. legends). There are no details of the shRNAs to ADAM8 in the manuscript.

We have amended and clarified the legends to Figures 5 and 6 as requested. We apologize for omitting the sequence of ADAM8 shRNA. It has now been added to the Material and Methods (p. 19).

p.10 ADAM8 and the 'release' of pro-angiogenic factors. Is there any evidence that this process involved the proteolytic function of ADAM8 (as cited in the Introduction)? This is not discussed here or in the Discussion but is a relevant issue and needs to be addressed.

As discussed above for Referee #1, this is an interesting question that had not been previously addressed in the literature. In response to your question, we tested the ability of ADAM8 proform vs remnant form (lacking the metalloprotease domain) to release VEGF-A. VEGF-A levels in conditioned medium from the shADAM8 clone increased upon ectopic expression of full-length ADAM8 but not the remnant form. These data, which have been included in a new panel Fig 5I, suggest that ADAM8 proteolytic activity is required for VEGF-A release.

Fig.7A, activity of R&D MAB1301 against ADAM8 seems to be rather poor compared to the previous ADAM8 shRNA KD data. However, the amount of antibody administered was rather low. Was a dose study carried out? Did higher levels of MAB1301 show more efficient abrogation of tumour development? Is MAB1301 known to inhibit ADAM8 proteolytic activity?

We had originally planned on using the same dose that was successful in one of the initial tests of Herceptin in preventing tumor growth (1 mg/kg) (Baselga, Cancer Res 1998). Unfortunately, the actual dose was half of that (0.5 mg/kg) due to a production error by R&D Systems, which we only learned about after the experiment was completed. Importantly, the findings obtained with 0.5 mg/kg ADAM8 antibody were significant and similar to the data obtained with 0.3 mg/kg Herceptin antibody. As we were using a commercial antibody and not one that would go into the clinic, we decided against performing a dose study in favor of testing effects of the antibody on metastasis of pre-existing tumors. In this new experiment, a neoadjuvant protocol was used in which we initiated antibody treatment prior to surgery and selected a 3-fold higher dose of 1.5 mg/kg (based on the comparison with Herceptin). Thus, tumors were grown to approximately 60-80 mm^3 (n=8 or 9 for isotype control and anti-ADAM8 antibody, respectively) when angiogenesis was already developed and then mice were injected with antibody twice in a span of one week prior to tumor resection. An additional dose was given on the day of tumor resection. There was a small but not significant decrease in tumor weight with ADAM8 antibody vs isotype-matched control IgG2B. Antibody treatment was continued twice weekly for an additional 5 weeks and the

effects on metastases assessed. A profound decrease in frequency of metastases to the brain and lungs was seen with the ADAM8 antibody treatment. While metastases to both the lungs and brain were seen in 8/8 and 7/8 mice, respectively in the control animals, only 2/9 and 1/9 anti-ADAM8 mice displayed metastases to these organs. These new data, which further validate ADAM8 as a therapeutic target, have been included in new panels Fig 7F and 7G.

As for mechanism of action, the ADAM8 antibody was made against the entire ectodomain and detects an epitope in the metalloprotease and disintegrin domains, but little was known about its effect on the protease activity of ADAM8. In response to your question, we have now shown that the ADAM8 antibody reduces VEGF-A release in the tumors *in vivo* (Fig 7E) and ADAM8 protease activity *in vitro* (Supplementary Fig S9).

Referee #3

ADAM8 is a transmembrane protein that belongs to the ADAM (a disintegrin and metalloprotease) family. Here, the investigators report that ADAM8 protein is expressed in 34% triple-negative breast cancers (TNBCs) (versus not detectable in normal breast tissue) and its level correlates with disease free and overall survival. The investigators provide evidence that ADAM8 promotes angiogenesis and metastasis in mouse xenografts.

This is a compelling and well-crafted manuscript that investigates the mechanism of ADAM8 signaling in breast carcinogenesis. The manuscript is well organized and experiments well designed and described. The investigators provide evidence that ADAM8 is an important regulator of poor prognosis breast cancer.
Suggestions for strengthening the manuscript:

1. It would be useful to know the frequency of ADAM8 expression in breast cancer subtypes other than TNBC.

The expression of *ADAM8* mRNA in the different breast cancer subtypes is shown in Fig 1D. If we were to show the data based on frequency of expression, we would have to select an arbitrary cut-off point. Depending on the cut-off point selected, the positivity rates could vary anywhere from low to high. Our presentation format (Fig 1D) is generally agreed to be the best way for representing this type of data.

2. The investigators postulate a role for ADAM8 in TNBC. Survival curves in Figure 1.F and G should be stratified for TNBC as well as for all breast cancers. This analysis would better differentiate whether ADAM8 predicts poor prognosis (as opposed to ADAM8 being expressed in TNBC more frequently and TNBC carrying a poor prognosis relative to other breast cancer subtypes).

We considered doing this analysis but as TNBC constitutes only approximately 15% of all breast cancers, the van de Vijver dataset only includes a small number of TNBC samples (i.e., 41). It would not be appropriate to use such a small number of patients to identify a possible prognostic effect. In response to your question, however, we re-ran the analysis after removing the TNBC samples from the total population and we found that high *ADAM8* expression was still significantly associated with a poorer prognosis in the remaining breast cancer patients (overall survival using 75th percentile cutoff in the dataset minus basal samples: $P = 0.003$, HR = 2.41 CI = 1.36-4.27).

3. The reason(s) for differences in the migration of the pro-form of ADAM8 in Hs578T is speculative and should be presented as such.

We have removed the comments in the Discussion section on the reason for the differences in mobility of ADAM8 in Hs578T cells.

4. The statement that "... ADAM8 contributed to the maintenance of a mesenchymal phenotype of TNBC cells in 3D-culture.", in the 1st paragraph of the discussion, is poorly supported by the data presented.

We have removed the term “mesenchymal” phenotype from the manuscript and use “invasive” phenotype, which is consistent with the observed role of ADAM8 in TNBC.

5. The Discussion is well written but would be improved by additional focusing (25-35% reduction).

The Discussion section has been shortened, as requested.

In summary, despite advances in breast cancer treatment, TNBC frequently progresses to invasive carcinoma with fatal metastatic spread particularly in younger women. Overall, our studies present compelling evidence that ADAM8 represents a promising novel target for treatment of patients with TNBC and suggest that an antibody-based therapy would be successful.

We thank the reviewers for their suggestions and comments and feel that our revised manuscript is substantially improved with these changes. We hope that it will now be in an acceptable form for publication in EMBO Molecular Medicine.

Your consideration of our manuscript is greatly appreciated.

3rd Editorial Decision 04 November 2013

Thank you for the submission of your revised manuscript to EMBO Molecular Medicine. We have now received the enclosed reports from the Reviewers that were asked to re-assess it. As you will see the reviewers are now supportive and I am pleased to inform you that we will be able to accept your manuscript pending the following final amendments:

1) As per our Author Guidelines, the description of all reported data that includes statistical testing must state the name of the statistical test used to generate error bars and P values, the number (n) of independent experiments underlying each data point (not replicate measures of one sample), and the actual P value for each test (not merely 'significant' or 'P < 0.05').

2) We would need a short list (up to 5) of bullet points that summarize the key NEW findings. The bullet points should be designed to be complementary to the abstract and will be used online in our new platform (coming January 2014).

Please submit your revised manuscript within two weeks. I look forward to seeing a revised form of your manuscript as soon as possible.

***** Reviewer's comments *****

Referee #1 (Comments on Novelty/Model System):

The revision is much better than the original & these data are worthy of publication.

Referee #1 (Remarks):

The authors have adequately answered our questions.

Referee #3 (Remarks):

In the revision of the manuscript "ADAM8 expression in invasive breast cancer promotes tumor dissemination and metastasis" by Romagnoli et al. the concerns of this reviewer were adequately dealt with. The authors show that high ADAM8 correlates with poorer disease free and overall survival in breast cancer. Further, it is shown that ADAM8 may act by inducing the release of VEGF-A, and other angiogenic factors, and increase adhesion to endothelial cells through activation of beta-1 integrin. Fianlly, it is shown that knockdown of ADAM8 results in a loss of an "agressive" phenotype in vitro and decreased umor growth and metastasis in vivo.
